# Open-Source Platform for Adjustable Training Regimes in Freely Moving and Head-Fixed Mice

**DOI:** 10.1523/ENEURO.0459-25.2026

**Published:** 2026-03-10

**Authors:** Michael D. Crespo, Sabrina M. Vaillancourt, Elizabeth A. Goldstein, Maria R. Broderick, Garrett T. Neske, Sandra J. Kuhlman

**Affiliations:** ^1^ Department of Biomedical Engineering, University at Buffalo, Buffalo, New York 14260; ^2^ Department of Physiology, University at Buffalo, Buffalo, New York 14203; ^3^ Neuroscience Program, University at Buffalo, Buffalo, New York 14203

**Keywords:** 2-photon imaging, calcium imaging, closed-loop, decision-making, perception, skill acquisition

## Abstract

Molecular tools available for rodent research enable detailed interrogation of the neural cell types and circuits that give rise to perception and decision-making during complex behaviors. To take full advantage of these molecular tools and successfully define causal relationships between neural function and overt actions during learning, there is a need for low-cost behavioral platforms with inherent flexibility in the implementation of task details. We present a behavioral platform capable of executing both head-fixed and freely moving task designs. The platform incorporates a user-interactive GUI that allows parameters to be adjusted online, during an acquisition session. Task metrics and performance indicators are acquired and organized into a standardized output, enabling single users to quickly master data analysis across a variety of task designs. To demonstrate the flexibility of the platform, mice of either sex were trained in two discrimination tasks: a head-fixed two-choice task as well as a freely moving operant conditioning task. Furthermore, we demonstrate that the platform can be used to show that mice harboring a mutation associated with autism spectrum disorder are able to perform a basic visual discrimination task in freely moving conditions. The presented work demonstrates the integration of multiple external devices to record task-related variables in a synchronized manner. As a result, the platform provides a valuable tool for affordable and reproducible investigation of behavioral decision-making as well as the neural basis underlying cognitive processes in health and disease.

## Significance Statement

An open-source, low-cost solution to implementing complex rodent behavioral training paradigms in head-fixed and freely moving conditions is presented. The platform offers flexibility in that it can be coupled to any number of external devices, each with unique sampling rates, task structures are composed of modular epochs, and on-the-fly adjustments to parameters critical for optimizing training can be made. Acquired data are organized into a standardized format, facilitating data visualization and analysis following updates to external devices, troubleshooting, and across a wide variety of behavioral task designs, including closed-loop systems.

## Introduction

Utilizing external stimuli to guide behavioral decisions is a constant and necessary part of survival. To study the neural basis of perception and decision-making in animal models, behavioral paradigms are used to assess perception and decision outcomes (Fetsch, 2016). Using cell type-specific molecular tools available in rodents, it is possible to delineate the neural circuit mechanisms that govern sensory-guided behavior during learning and development. A single study often incorporates multiple variations of a task, including altering parameters of sensory stimuli, reward contingencies, reward probability, and stepwise shaping, among others (Bari et al., 2019; Reinert et al., 2021). To capture natural dynamics in neural activity and behavior, freely moving tasks are preferred. For example, when high throughput is desired, head-fixation stress is a confounding factor (Juczewski et al., 2020), home-cage training is desired (Audette et al., 2019), or when more naturalistic behaviors are the focus (You and Mysore, 2022; Shir et al., 2023; Skyberg and Niell, 2024), freely moving tasks are required. Behavioral tasks performed by head-fixed animals are used when study designs require precise and repeated longitudinal monitoring or manipulation of neural activity, incorporate recording modalities capable of collecting data from large populations of neurons, or require accurate spatiotemporal control of environmental variables such as stimulus size, reward delivery, and scheduled delays. Furthermore, technology for acquiring high-resolution imaging data using chronic miniature 2-photon microscopes (Zong et al., 2022; Wu et al., 2025) and wireless neurophysiological recordings (Kwon et al., 2024; Newman et al., 2025), as well as awake fMRI (Gutierrez-Barragan et al., 2022; Hike et al., 2025; Molhemi et al., 2025), is advancing. Thus, studies are increasingly incorporating both head-fixed and freely moving behaviors to reach research objectives (Glas et al., 2019; Zixiao et al., 2025). Yet, turn-key behavioral systems can be cost-prohibitive, particularly in cases in which multiple behavioral frameworks are incorporated into a single study. As such, there is a need for an open-source platform that facilitates the flexible implementation of behavioral tasks in a standardized format across a range of frameworks, including two-choice paradigms, Go/No-go paradigms, reversal learning, and operant conditioning during freely moving behavior (White et al., 2019).

**Figure 1.**
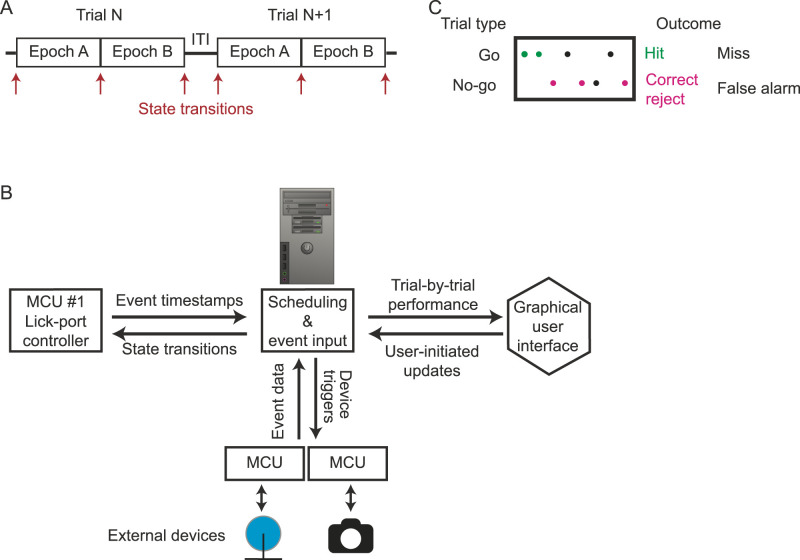
Components of the behavioral platform. ***A***, An example of state transitions that can be implemented within a session. Each Epoch can have several associated trial types, for example, rewarded or not rewarded. Reward size can be varied. Typically, an intertrial interval (ITI) occurs between the last Epoch of a trial (Trial N) and first Epoch of the next trial (Trial *N* + 1). ***B***, Logic of hardware and software connectivity. The computer program schedules trial types. The program receives event timestamps from microcontroller unit (MCU) #1 which serves as a hub controlling the lick-ports and interfaces with additional external devices, coupled to the program using additional MCUs. The program generates timestamps for event data from all additional MCUs. Trial-by-trial performance is viewable by the user through a graphical user interface (GUI). The GUI includes features that enable the user to update parameter settings within an ongoing session. State transitions are initiated by the computer program directly or after being initialized by events received from an MCU. ***C***, Example graphical user interface (GUI) for a Go/No-go experiment. The scheduled trial type is labeled by row, and all possible behavioral responses and the associated trial outcomes are labeled on the right.

To address this need, we created a low-cost, open-source behavioral platform useful for implementing an array of tasks and training regimes, including stepwise shaping in which elements of the final task are sequentially introduced to the subject. Here, we demonstrate the platform's flexibility by training mice in two different frameworks, each with a distinct shaping protocol: first, a two-choice visual discrimination task in which a head-fixed animal's own locomotion controls the speed of a visual presentation (closed-loop stimulus presentation) and second, a freely moving operant Go/No-go discrimination task. Importantly, additional data from external devices can be acquired and synchronized with the core task metrics, such as an eye tracking camera (Movie 1) as well as neurophysiological imaging and optogenetic stimulation. This is accomplished by a software program that aligns the data acquired from the various external devices with core task metrics on a trial-by-trial basis (Fig. 1, Table 1). Together, this platform allows a standardized approach to behavioral task generation that will provide efficient implementation and analysis of a wide variety of behavioral tasks, easily customizable for the specific question at hand and neural recording device used.

**Movie 1.** Video of mouse performing the closed-loop two-choice visual discrimination task, one example trial. Synchronization of task epochs, lick events, and output from an external device (the wheel, data are reported as velocity) is shown. Top, Velocity is coupled to visual flow. Middle, The distance traversed in the trial is indicated. Bottom, Locomotion on the wheel and lick events are indicated. [View online]

**Table 1.** Output data structure with key variable fields described

Variables	Format	Dimensions	Description
TrialID	Double	1 × 1	Trial number, typically one through the total number of trials
TrialType	String	1 × 1	In this example there are two trial types, vertical or angled
NoOfTimestamps	Double	1 × 1	Total number of timestamps in a given trial
Timestamps	Double	Number of timestamps × 1	Timestamps from MCU#1, the lick-port controller, aligned to one chosen epoch. In this example, timestamps are aligned to the “Grating” epoch
isLickLeft	Logical	Number of timestamps × 1	1 indicates the timestamp is a “lick left” event
isLickRight	Logical	Number of timestamps × 1	1 indicates the timestamp is a “lick right” event
isGratingStart	Logical	Number of timestamps × 1	1 indicates the timestamp is the epoch start event
isRewardZoneEntry	Logical	Number of timestamps × 1	1 indicates the timestamp is the reward zone entry event
isRewardTriggered	Logical	Number of timestamps × 1	1 indicates the timestamp is the reward triggered event. This is equivalent to the time the reward valve was opened
isGratingEnd	Logical	Number of timestamps × 1	1 indicates the timestamp is the epoch end event
isManualOpen	Logical	Number of timestamps × 1	1 indicates the respective timestamp in which reward was manually dispensed (e.g., not triggered by the subject's behavior)
encoderData_combined	Double	Number of samples × 2	Distance in corridor in cm (column 2) and time (column 1) of sample taken relative to grating onset. Negative values represents time until grating epoch onset and negative distance represents distance left to travel in approach epoch until grating onset starts
Velocity	Double	Number of samples × 2	Velocity is calculated for each data point as the difference between the current position in corridor relative to the previous position, divided by the difference in time between those two samples. Column 1 is same time as encoderData_combined, column 2 represents velocity in cm/s. First data point will always be NaN
frameData_combined	Double	Number of frames × 2	Records the frame number in the resulting video (column 2) and the time frame capture began (column 1), relative to grating onset
pupilRadius_combined	Double	Number of frames × 2	Displays the calculated pupil radius of the frame reported in frameData_combined (Column 2) and the time frame capture began (column 1), relative to grating onset. Time is same as frameData_combined

The acquired data are organized by trial and saved in a MATLAB “struct” format.

## Materials and Methods

### Animal welfare

All animal procedures were performed in accordance with the University at Buffalo's animal care committee's regulations.

### Behavioral platform

The behavioral platform couples flexible software with modular hardware to allow for the integration and organization of multiple aspects of behavior across a wide array of tasks (Fig. 1). A complete list of parts used, a detailed protocol of hardware assembly and software installation, all code used, as well as relevant information for implementation are available freely (https://github.com/Mdcrespo/BehavioralPlatform).

The platform is designed for use cases in which animals are expected to perform multiple trials within a session. Data acquisition and output is formatted in a trial-based structure. The trial structure itself is flexible and can be composed of a sequence of epochs. Epochs represent subsections of a trial which contain distinct stimuli or task rules (Fig. 1*A*). For example, in the first demonstration task, mice enter an approach zone (Epoch A) prior to encountering the target stimuli (Epoch B). The scheduling of trial types (e.g., Go vs No-go in the case of a single lick-port paradigm, or left vs right in the case of the two lick-port paradigm) and the timing of state transitions within a session are controlled by the computer program (Fig. 1*B*). After the session is completed, acquired data are aligned on a trial-by-trial basis and stored in a MATLAB “struct” variable with multiple fields (Table 1).

Notably, a graphical user interface (GUI) accepts callbacks at the end of each trial, allowing the experimenter to alter parameters of their choosing during an ongoing session (Table 2), thereby affording additional flexibility to address behavioral side biases or facilitate shaping in the early stages of training (Fig. 1*B*,*C*).

**Table 2.** Task parameters allowing for within session modulation

Task parameter	Description	Activation criteria	Behavioral task related to parameter
Auto water delivery	Releases a water reward shortly before the end of stimulus presentation if mouse does lick correct side	Manual, can be toggled to activate within that trial	Head-fixed task
Xleft	Allows 3 rewards to be collected on vertical trials (rewarded through left port)	Manual, can be toggled to activate within that trial	Head-fixed task
Xright	Allows 3 rewards to be collected on angled trials (rewarded through right port)	Manual, can be toggled to activate within that trial	Head-fixed task
Open valve	Immediately releases water. If in the grating corridor, water is released from the port associated with the stimulus, otherwise released randomly from one port	Manual, can be triggered to instantly activate once	Head-fixed task
AutoClicker	Toggles whether uncoupled relay causes intermittent clicking sound	Manual, can be toggled to activate within that trial	Head-fixed task
Consecutive trial limit	Prevents same stimulus being presented more than 3 times consecutively	Automatic, alters next trial stimulus when trials schedule is being ordered at start of session	Head-fixed task
Auto water delivery	Releases a single water reward shortly before the end of stimulus presentation if mouse does not activate water reward through correct behavioral response	Manual, can be toggled to activate within that trial	Head-fixed shaping
Open right valve	Immediately releases water on the right	Manual, can be triggered to instantly activate	Head-fixed shaping
Open left valve	Immediately releases water on the left	Manual, can be triggered to instantly activate	Head-fixed shaping
AutoClicker	Toggles whether distractor relay causes intermittent clicking sound	Manual, can be toggled to activate within that trial	Head-fixed shaping
Side-bias deterrent rule	Prevents water from being released from one side until other side is interacted with	Automatic, engaged if consecutive interaction threshold is met, without need of user	Head-fixed shaping
Open valve	Immediately releases water from the lick-port	Manual, can be triggered to instantly activate once	Freely moving task and shaping
Consecutive trial limit	Prevents same stimulus being presented more than 10 times consecutively	Automatic, alters next trial stimulus when trials schedule is being ordered at start of session	Freely moving task and shaping

Parameters are either automatically modulated based on the responses of the mouse or manually modulated within the GUI by the researcher during the session.

In addition to scheduling trial types and gating the timing of state transitions, the program is capable of continually sampling event data transmitted from one or more microcontroller units (MCUs), at a sampling frequency up to 300 Hz. The platform is designed to use Arduino microcontrollers. Either event timestamps from MCU #1 or event data from external devices can be integrated by the program; the program then triggers a state transition. For example, in the provided closed-loop example, the distance traveled on the wheel is transmitted to the program, and then the program triggers the state transition between Epoch A and Epoch B once the accumulated distance surpasses transition threshold.

It is worth noting that in this specific example of the wheel, the rotary encoder is directly connected to an MCU, as such the encoder-MCU ensemble essentially serves as a data acquisition device. However, in most cases the external acquisition device will have its own independent acquisition system, with its own sampling rates that may exceed 300 Hz, such as when recording electrophysiological or imaging signals. In such cases, event data is not necessarily the raw data acquired by the external device, rather, user-defined data useful for aligning timestamps between the external device and the program. Therefore, our system is compatible with acquiring data at kilohertz frequencies (or higher), typical of electrophysiology experiments.

The system inherently uses two clocks, one at the level of the MCU #1 and one at the level of the computer program. The MCU #1 clock is used for session-wide recording of state transition timing, as well as recording lick event and valve event timing. The computer program clock is used for timestamping event data streamed from external devices relative to epoch onset. This design enables data from external devices to be aligned to one clock, the computer clock. As such, the addition of any number of external devices (each with different sampling rates and/or capture rates) does not require incorporating more clocks or new timestamping mechanisms into the program.

In terms of benchmarking the time difference between the two clocks (MCU #1 and the computer program), we found that for a given epoch the difference between the MCU #1 timestamped duration and the program timestamped duration is an average of 0.886 ms and not more than 3 ms. In practice this means, on average, timing discrepancies between reward-related lick events and external device timing is <1 ms and does not exceed 3 ms.

At its core, the platform is based on three components: (1) a MATLAB program run on a Windows-based operating system, capable of receiving event input and scheduling trial types and state transitions, (2) a microcontroller dedicated to controlling the lick-ports, and (3) a GUI that displays trial-by-trial performance capable of sending user-initiated updates to preselected parameters such as manual reward dispensing. In addition to these three core components, external devices can be coupled to the system via incorporating additional MCUs. External devices can include devices to present sensory stimuli, such as a computer screen to display visual stimuli, speakers to present auditory stimuli, or air puff units for the delivery of somatosensory or olfactory stimuli. In the closed-loop task example provided, four external devices are used. A description of how the acquired behavioral data are stored in this example task is provided in Table 1. Note, the number of digital I/O channels on the microcontroller sets the upper limit of how many lick-ports could be controlled from MCU #1. If using Arduino mega, that number is 51.

### Animal housing and preparation

To generate wild-type mice for experiments, either homozygous or hemizygous tetO-GCaMP8s mice (Jackson Laboratories, stock number 037717) were bred with hemizygous CamK2a-tTA mice (Jackson Laboratories, stock number 007004). To generate haploid mutant mice, heterozygous mice expressing C-terminal (exon 21) deleted Shank3 (Jackson Laboratories, stock number 018398) were bred to homozygosity, and then homozygotes were mated with wild-type mice to produce haploid mice for experiments. Mice were housed in groups of 2–3 per cage unless behavioral aggression warranted single-housing. The light/dark cycle was set to 12 h lights-on/lights-off, where lights-off is referred to as zeitgeber (ZT) 12. For mice performing the two-choice visual discrimination task, once the mice reached the age range of approximately postnatal days (P) 30–P40, they underwent surgery while under the effect of isoflurane anesthesia. This involved securing a custom-made stainless steel bar onto the right side of the skull using dental acrylic (Feese et al., 2018), which was used to head-fix the mouse for behavioral training (Kowalewski et al., 2021). The mice were allowed to recover from the procedure for at least 3 d with *ad libitum* food and water access, before the water restriction occurred. Water restriction was initiated prior to shaping and once placed on restriction was not removed from water restriction until the end of the experiment. Mice received 750 µl of water per day while on restriction. The weight of the mouse was recorded daily. Shaping began once the mice reached weight stabilization. Weight was considered stabilized once daily weight change was <0.1 g for 3 consecutive days, and generally weight was stabilized within 8–10 d; mice were on water restriction for at least 8 d prior to starting the first shaping task in all cases. Mice were maintained on water restriction for up to 2.5 months. In the case mice did not earn ≥750 µl during a session, they were supplemented such that their water intake was 750 µl for the day.

### Behavioral tasks and training schedules

Male and female mice were trained in one of two unique visual behavioral tasks: a closed-loop two-choice visual discrimination task (*n* = 6 mice, 2 male 4 female) and a freely moving operant conditioning visual discrimination task (*n* = 2 mice, 1 male 1 female). Behavioral experiments commenced at approximately ZT15, during the lights-off phase.

### Closed-loop two-choice visual discrimination task

#### Behavioral apparatus

During behavior, mice were head-fixed onto a stainless steel bar positioned over a cylindrical wheel where wheel locomotion was recorded with an optical quadrature rotary encoder (US Digital) coupled to an Arduino Mega (MCU #2, External device). The wheel and the screen were calibrated so that 1 cm of movement on the wheel resulted in 1 cm of movement of the shown stimulus in the direction of wheel movement. The screen (Dell; 30″, 2,560 × 1,600 resolution) was positioned 25 cm away from the mouse in front of the right eye, angled at 50° with respect to the midline of the animal (Extended Data Fig. 2-1).

**Figure 2.**
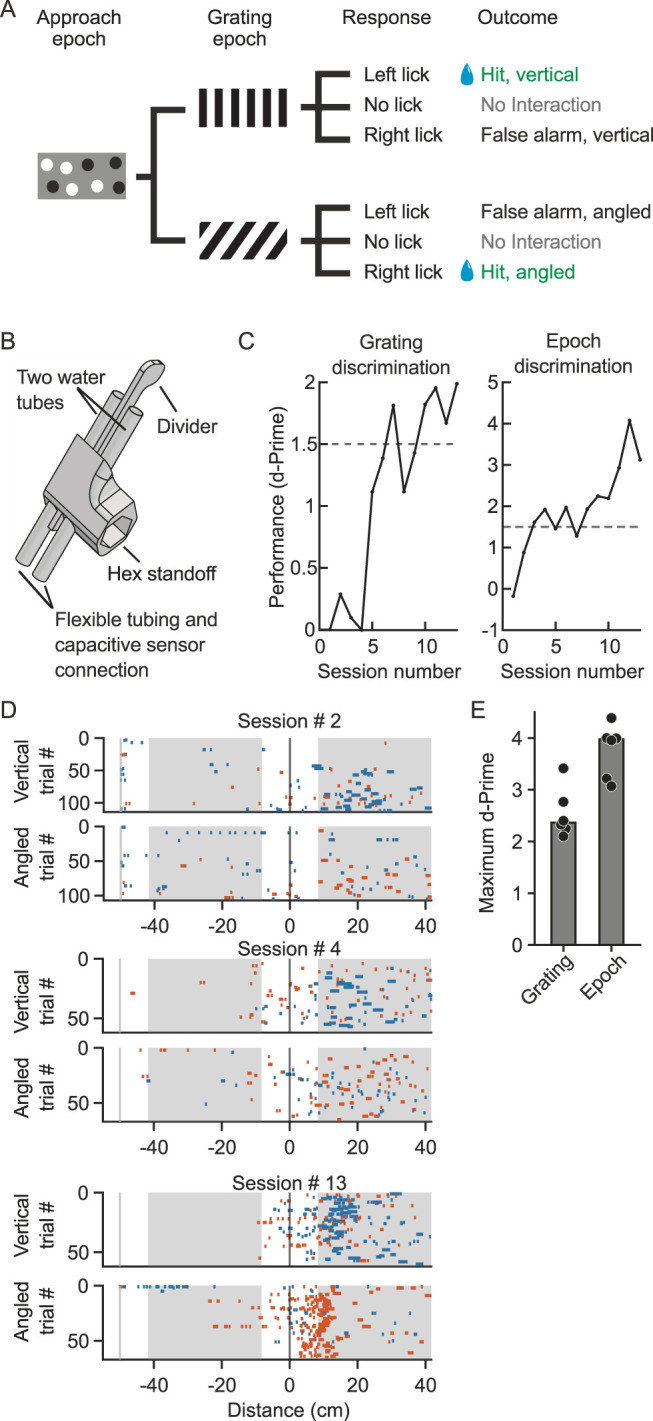
Implementation of a head-fixed closed-loop two-choice visual discrimination task. ***A***, Schematic of trial structure, including two Epochs, Epoch A, “Approach,” and Epoch B, “Grating.” The computer program schedules the trial type, either a vertical grating in which the animal must lick to the left to receive a reward, or an angled grating in which the animal must lick right to receive a reward. All possible behavioral responses and the associated trial outcomes are indicated. Rewarded outcomes are indicated with a blue water drop symbol. The state transition between the approach epoch and the grating epoch is initialized by an external device MCU, specifically the distance traveled on the running wheel, via a rotary encoder. Positioning of the external devices relative to the head-fixed subject are depicted in Extended Data Figure 2-1. ***B***, Schematic of the 2-port holder and divider in relation to the two water tubes. The divider separates the two water tubes to ensure that mice contact only one of the two water tubes during a single lick trajectory. The holder contains an empty cavity where a hex standoff can be attached (e.g., Digikey, 36-1921-ND). Flexible tubing and capacitive sensors are connected to the water tubes, the subject collects water at the other end of the water tubes. The distance of the divider relative to the holder and the two lick-ports can be manually adjusted. Further distance details are depicted in Extended Data Figure 2-2. ***C***, Behavioral performance across 13 sessions in an example wild-type mouse. The use of a two-port system allows two different discriminations to be assessed, Epoch discrimination and Grating discrimination. Note, performance on Epoch discrimination crossed a threshold of 1.5 (dashed line) prior to Grating discrimination. ***D***, Raster plots of lick responses for three different selected sessions in an example mouse. Lick times were aligned to Grating Epoch entrance and sorted post hoc into trial type. Blue, licks to the left; orange, licks to the right. Shading indicates the distance over which the first lick was scored for the calculation of *d*-prime shown in ***C***, Epoch discrimination. ***E***, Six of seven mice learned the two-choice discrimination task; six mice achieved a *d*-prime grating discrimination performance of at least 2, and a *d*-prime epoch discrimination of at least 3. One mouse failed to walk on the wheel, so shaping was discontinued. Data points are individual mice, the bar height indicates the median across six mice.

A custom 3D-printed lick-port holder (CAD files provided; https://github.com/Mdcrespo/BehavioralPlatform) was used to hold two lick-ports in parallel (Fig. 2*B*). Lick-ports were built from a 0.094-inch-outer diameter stainless steel water tube with a 0.02-inch-wall thickness (6100K441, McMaster-Carr) connected to a capacitive sensor (SparkFun, AT42QT1011) at the water supply end of the water tube. Tongue contacts on the water tube are sufficient to activate the capacitive sensor. Each assembled lick-port functions to both detect lick events and deliver liquid rewards. When positioned in parallel, the water tubes were separated by 5.0 mm and were placed in front of the midline of the mouse's snout, such that each water tube was 2.25 mm away from the mouse's snout. To prevent licking both water tubes at the same time, a custom-printed divider was placed between the two water tubes and was positioned ∼2.3–3.0 mm from the mouse. Spacing of these elements is depicted in Extended Data Figure 2-2. Each lick-port reported lick event detection to MCU #1, which recorded the timestamp of each detection. Water rewards were delivered by gravity using a 3-port solenoid valve that opened for a defined period following a rewarding event (Lee Company; LHDA1233115H). The volume of the water delivered per reward was controlled by the duration that the solenoid valve was open and was set to release ∼6 µl per triggered valve opening. A relay was used to gate the solenoid (4411-TS0010D-ND, Digikey). Each solenoid valve was connected to their respective lick-port via a 0.125-inch-outer diameter plastic tube with a 0.03125-inch-wall thickness (E-3603, Tygon).

The relay generated a clicking sound when activated. To ensure that mice did not associate the sound of the relay clicking with the water reward on trials where water was automatically presented, a second relay was used to replicate the clicking sound and was set to trigger at a random interval between 0.5 and 3 s. This relay was solely used to uncouple the clicking noise from water reward alone and would not administer water and was connected as an external device. Images of the eye were recorded at 10 Hz (Teledyne FLIR, FL3-U3-13E4C-C) with the pupil illuminated by an infrared (IR) light-emitting diode (850 nm). Frame timing, as well as timing of the uncoupled relay, was controlled by another Arduino Uno (MCU #3, External device, not shown in Fig. 1*B*). Stimulus presentation for all tasks was controlled using Psychophysics Toolbox (PTB-3; http://psychtoolbox.org) in MATLAB (MathWorks). Encoder and frame acquisition were synchronized and timed within MATLAB and aligned to epoch onset within each trial. This record was saved as a directory containing individual MATLAB variables with the saved information for each trial, separated by epochs. Lick events and reward valve release are monitored using an Arduino Uno (MCU #1). The session-wide timing of these events and state transitions initiated by the computer program were recorded by MCU #1. This record was saved as a single text file.

The cost of implementing this system, with three Arduinos, one computer, and four external devices, was less than $3,000.

#### Acclimation to task environment

The goal of shaping was to encourage the mouse to walk (necessary, given the task is closed-loop), and to teach the mouse that water could be released from the lick-port. Each mouse was acclimated to the trial structure and testing environment by being placed on the wheel, with a blank gray screen on the monitor. For each trial, once the mouse traveled 20 cm, the screen randomly switched to fully black or white for 6 s. During this interval, if the mouse licked one of the two lick-ports, a water reward was released from that lick-port. During the first session, a single water reward would automatically be released randomly from one of the two lick-ports 2 s after stimulus change to encourage the mouse to lick the lick-ports. Once the mouse began to lick the lick-ports, the automatic water option was turned off within the session. The determination to activate or deactivate the automatic water option was made by the observer.

From pilot data, we found that two mice quickly exhibited a side-bias. Therefore, we implemented a side-bias deterrent rule as follows: If the mouse licked the lick-port on the same side 3–8 times in a row (criteria threshold randomized trial-by-trial), water would no longer be released from that lick-port (the consecutive side), and water would automatically be released from the other lick-port 2 s after stimulus change on the subsequent trial. Water would not be released on the consecutive side until a trial occurred where the mouse manually triggered water release from the other lick-port. This side-bias rule was coded into the program and executed automatically without requiring further observer input.

Once the mouse was able to collect at least half of the daily water within a single session, the mouse was moved to the two-choice discrimination task.

#### Two-choice discrimination task

Mice were trained in the task 5–7 d a week. For each session, the mouse performed a series of trials, with each trial containing two distinct epochs, followed by a fixed 2 s intertrial interval in which an isoluminant gray screen was presented (Fig. 2*A*). The first epoch, referred to as the approach epoch, was 50 cm in length and consisted of a gray background with a series of randomly placed, nonrepeating white and black circles, ranging between 10 and 30° in diameter. The locations of the circles ensured that no two segments of the approach epoch display an identical image on the monitor. Once the mouse traveled through the approach epoch, the screen abruptly transitioned to the second epoch of the task. Within the second epoch (grating epoch), also 50 cm in length, one of two grating stimuli were presented, either a 0° vertical grating (left licks rewarded) or a 135° diagonal grating (right licks rewarded), both with a spatial frequency of 0.04 cycles/°. A 2-port water delivery system was designed to facilitate training (Fig. 2*B*). Both stimuli had a 50% likelihood of appearance; however, neither stimulus was presented for more than three consecutive trials. Traveling through the grating epoch ended the trial.

During the grating epoch, once the mouse traveled a short distance (8.33 cm), a water reward (∼6 µl) was able to be triggered by the mouse licking. On a vertical grating trial, licking the left lick-port resulted in one drop of water reward from the left; licking the right lick-port on an angled grating trial resulted in one drop of water reward from the right. In each of these cases, the trial was considered a correct trial. On trials where the mouse did not lick (no-interaction trial) or on trials in which the mouse licked the incorrect side (error trials), a water reward was automatically delivered from the lick-port associated with the presented stimulus shortly before exiting the grating epoch (41.67 cm). This automatic water delivery option was manually set by the observer before session onset. If the mouse exhibited a side-bias toward one of the two lick-ports on any given session, during each subsequent session the mouse was able to collect three rewards on trials where the water was delivered from the nonpreferred side, until the bias was reduced. Bias correction was manually implemented by the observer for a given session if a bias was observed (see “Acclimation to Task Environment”) and the mouse exhibited significant discrimination between the two epochs of the task. Each session ended once the mouse collected 125 rewards (∼750 µl) or after 90 min passed since session onset.

#### Performance and side-bias analysis

Performance was calculated for two distinct aspects of the task. Behavioral *d*-prime (*d′*) was used to calculate performance for both aspects (Eq. 1):

$$d^{\prime }=z_{\mathrm{Hit}\;\mathrm{Rate}}-z_{\mathrm{False}\;\mathrm{Alarm}\;\mathrm{Rate}},\;$$

where *z* was the inverse of the standard normal cumulative distribution function.

For classifying discrimination between the two grating stimuli presented during the grating epoch, grating discrimination, hit rate was calculated as the proportion of vertical trials for which the mouse correctly licked left within the reward zone of the grating epoch (trials scored as “Hit, vertical”) out of the total number of vertical trials scored as “Hit, vertical” or “False alarm, vertical.” False alarm rate was calculated as the proportion of angled trials for which the mouse incorrectly licked left within the reward zone of the grating epoch (trials scored as “False alarm, angled”) out of the total number of angled trials scored as “Hit, angled” or “False alarm, angled.” Trials in which the mouse did not lick within the reward zone of the grating epoch were not considered for classifying grating discrimination.

For classifying epoch discrimination, hit rate was calculated as the standardized proportion of trials in which the mouse interacted with either lick-port within the center 2/3 of the grating epoch. False alarm rate was calculated as the standardized proportion of trials in which the mouse interacted with either lick-port within the center 2/3 of the approach epoch.

Mice were considered to have learned each aspect of the task on the first session in a three-session window in which at least two sessions had a corresponding *d′* >1.5 (Fig. 2*C*). In general, mice first attained expert status discriminating between the two epochs, before later exhibiting expert level discrimination between the two grating stimuli within the decision epoch (Fig. 2*D*).

Bias was calculated as an index, referred to as the side-bias index (Eq. 2):

$$\;\mathrm{index}=-|z_{\mathrm{Hit}\;\mathrm{Rate}}+z_{\mathrm{False}\;\;\mathrm{Alarm}\;\mathrm{Rate}}|/2,\;$$

where *z* was the inverse of the standard normal cumulative distribution function.

**Figure 3.**
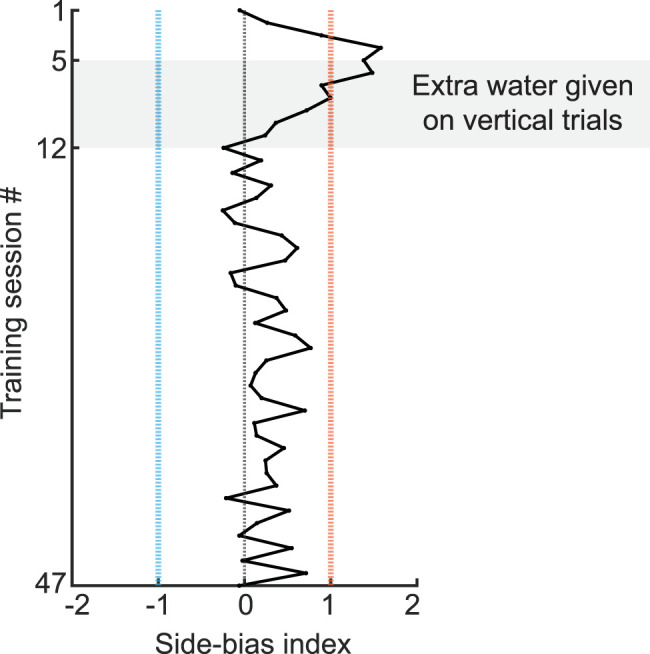
Identification and persistent correction of side-bias during the two-choice discrimination task. Example of licking bias to the right side (orange, side-bias index >1). Extra water was delivered during trials in which the vertical stimulus was presented (left side) for a total of 6 sessions (session numbers 5–12). Reward size was re-equalized on session number 13. Note, the side-bias index did not exceed a value of 1 after the reward size was re-equalized (session numbers 13–47).

Hit rate and false alarm rate were calculated as in grating discrimination. An absolute value >1 signified that the number of lick events between the two sides likely differed by at least one standard deviation. As such we chose an absolute value of 1 as the criteria threshold for implementing bias correction. Positive values indicated a bias toward licking to the right, negative values indicated a bias toward licking to the left. Bias was considered reduced after at least two sessions in which the side-bias index was <0.25, and the reward size was then re-equalized to one drop of water for both sides (Fig. 3).

### Freely moving operant conditioning visual discrimination task

#### Behavioral apparatus

**Figure 4.**
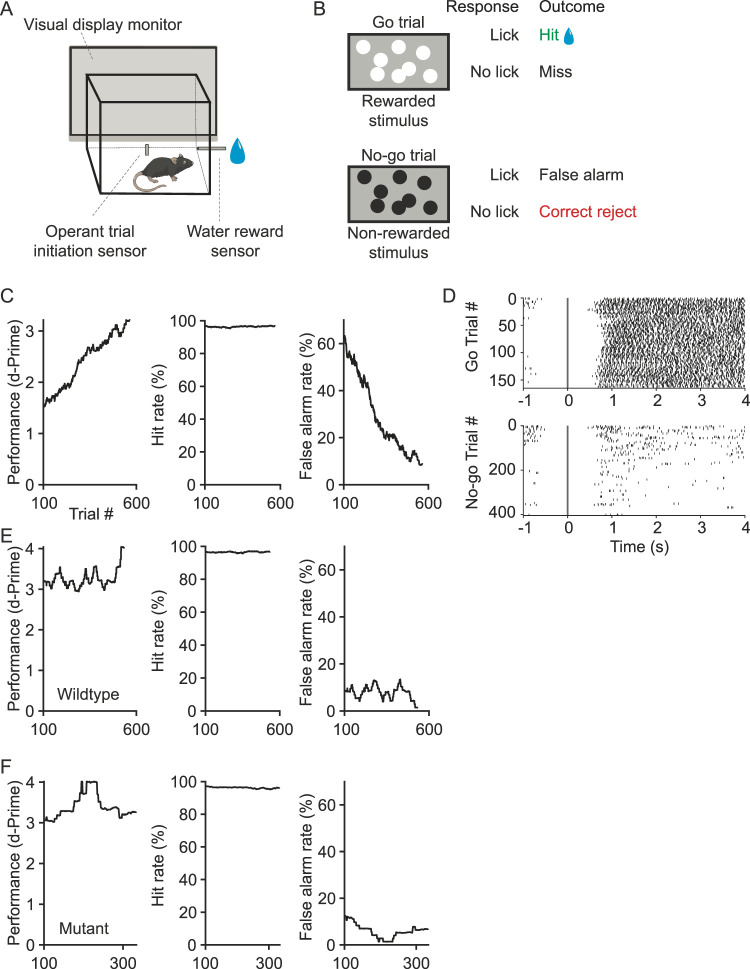
Implementation of a freely moving operant conditioning discrimination task. ***A***, Schematic of environment for freely moving behavior. ***B***, Schematic of trial types, all possible behavioral responses and the associated trial outcomes are indicated. ***C***, Performance of an example wild-type mouse during the first training session after shaping was completed. A sliding window of 100 trials within the session was used to calculate performance (*d*-prime), hit rate, and false alarm rate. Increased performance was associated with a decrease in the false alarm rate. ***D***, Raster plots of lick events for the training session shown in ***C***. Lick times were aligned to the time that the stimulus epoch was self-initiated, sorted into Go and No-go trials post hoc. Stimulus probabilities: Go, 0.3, and No-go, 0.7. Density of lick events on No-go trials decreased with increasing trial number. ***E***, ***F***, Both wild-type and mutant mice maintained a low within-session false alarm rate after several sessions of training.

Mice were placed within a 15.2 × 15.2 × 15.2 cm plastic behavioral chamber (Brightroom). A screen (Dell; 20″, 1,600 × 900 resolution or Miktver; 10.5″, 1,920 × 1,280) was placed 9 cm from one wall of the behavioral chamber. To allow the mouse to self-initiate trials, the trial initiation sensor, a single metal rod connected to a single capacitive sensor (SparkFun, AT42QT1011), was placed in the middle of the chamber from the floor, 0.5 cm away from the wall which displays the stimulus. To detect lick events and deliver water rewards, a single lick-port, a 0.094-inch-outer diameter stainless steel water spout with a 0.028-inch-wall thickness (6100K451, McMaster-Carr) connected to a single capacitive sensor (SparkFun, AT42QT1011), was inserted into the wall right of the stimulus display wall, placed 1 cm from the floor and 2 cm from the corner between the display wall and the wall with the lick-port attached (Fig. 4*A*). Lick event and trial initiation event detection from the lick-port and the trial initiation sensor were each reported to MCU #1. Water rewards were delivered by gravity using a 3-port solenoid valve that opened for a defined period following a rewarding event (Lee Company; LHDA1233115H). The volume of the water delivered per reward was controlled by the duration that the solenoid valve was open and was controlled to release ∼4 µl per reward. Water was delivered from the solenoid valve to the lick-port for the mouse via a 0.125-inch-outer diameter plastic tube with a 0.03125-inch-wall thickness (E-3603, Tygon). Stimulus presentation for all tasks was controlled using Psychophysics Toolbox (PTB-3; http://psychtoolbox.org) in MATLAB (MathWorks).

The session-wide timing of lick events, valve release events, and state transitions initiated by the computer program were recorded by MCU #1. This record was saved as a single text file.

The cost of implementing this system, with one Arduino, one computer, and one external device, was less than $1,000.

#### Acclimation to task environment

The goal of shaping was to familiarize mice with the fact that water was sometimes available at the lick-port and to train mice to initiate trials by touching the trial initiation sensor. Each mouse was acclimated to the trial structure and testing environment using two sequential shaping tasks. Mice were placed in the environment with a blank gray screen on the monitor. During the initial shaping task, for each trial, after a small delay (0.5–3 s) 40 white circles of 50-pixel diameter were presented. Following stimulus presentation, if the mouse licked the lick-port, a water reward was released. Then, 0.5 s after reward delivery the stimulus would disappear, and the next trial would begin. Once the mouse was able to collect the daily water allotment within a 90 min session, in the next session on a subsequent day, the mouse was transitioned into a second shaping task.

The second shaping task follows the initial task with the addition of self-initiation. Within the task, the mouse must touch the trial initiation sensor to trigger stimulus presentation. Upon stimulus presentation, if the mouse licked the lick-port, a water reward was released. Then, 0.5 s after reward delivery stimulus would disappear, and the next trial would begin after a 0.5 s intertrial interval. Once the mouse was able to collect the daily allotment of water within a 90 min session, in the next session on a subsequent day, the mouse was moved into the luminance discrimination task.

#### Freely moving luminance discrimination task

Mice were trained 5–7 d a week. For each session, the mouse performed a series of trials, with each trial containing a prestimulus epoch and a decision epoch, followed by a 0.5 s intertrial interval. During the prestimulus epoch, a blank gray screen was presented, and the mouse was required to interact with the trial initiation sensor. Upon contact with the trial initiation sensor, the decision epoch immediately began with stimulus presentation of 40 circles of 50-pixel diameter of either high (white, Go) or low (black, No-Go) luminance. These circles are 6° when the mouse is in front of the trial initiation sensor and the position of the circles varied every trial. The stimulus was presented for 4 s, and the next trial began after a 0.5 s intertrial interval. The probability of each trial presenting a Go stimulus was 30%, with no trial-type having >10 consecutive presentations. On Go trials, after a 0.5 s delay, interaction with the water reward sensor would result in a water reward (∼4 µl; Fig. 4*A*). A brief intertrial interval is required for the GUI to update, and the minimum possible interval is 50 ms. In this example, an interval of 500 ms was used. Trial outcomes were as follows: If a lick occurred during a Go trial, reward was delivered, and the trial was counted as a hit; if no lick occurred, reward was not delivered, and the trial was counted as a miss. If the mouse licked during a No-go trial, no reward was delivered, and the trial was considered a false alarm; if no lick occurred, no reward was delivered, and the trial was considered a correct reject (Fig. 4*B*). Each session ended once the mouse collected 190 rewards (∼750 µl) or after 120 min passed since session onset.

#### Performance analysis

Performance was quantified as *d*-prime and hit/false alarm rate (Fig. 4*C*,*E*,*F*).

### Synchronization across devices

#### Two-photon microscopy

To synchronize behavioral data with imaging modalities such as two-photon microscopy, we provide code on how to accept a trigger originating from an example microscope system on the GitHub site noted above. Our example is for the miniaturized SuperNova-100 Two-Photon Microscope (Transvista). Briefly, the microscope is configured to send a high TTL signal during laser scanning and a low TTL signal when not scanning. The state of the TTL signal is read by MCU #1, at a sampling frequency of 300 Hz; thus the temporal precision of transmitting TTL transitions is within 3.3 ms. TTL transitions are timestamped and received by the program.

#### Pupillometry

The desired capture frequency (frame rate) of the camera is controlled by the MCU. Our example is for the Flea 3 Color USB 3.0 camera (Teledyne FLIR, FL3-U3-13E4C-C). Upon start of experiment, for each desired frame capture, a TTL pulse is sent from the MCU to both the capture camera as well as the master computer. The computer records the frame number relative to the overall video, as well as the time the frame was captured relative to epoch onset and at the same time frame generation begins.

For sessions where the pupil recordings were analyzed, each frame was first analyzed by a ResNet 18 deep learning model trained to classify frames in which the pupil is open, and the pupil is occluded. The model was first trained on 70 example open eye frames and 70 example occluded eye frames. These frames were taken from across two animals with frames from at least three sessions per animal used. The model utilized 15 open eye frames and 15 occluded eye frames for validation after every training epoch. The model was trained for 35 epochs. Upon epoch 35, both training and validation accuracy achieved 100% and training loss and validation loss reached 0.013 and 0.067, respectively. After training was completed, the model was tested on 15 new open eye frames and 15 new occluded eye frames, exhibiting a classification accuracy of 100%. Upon reaching 100% testing accuracy, the model was used to predict the class of each frame in each session. Training, testing, and predictions were performed utilizing the Torchvision ResNet model within python using Google Colab with the T4 GPU set as the primary compute unit.

**Figure 5.**
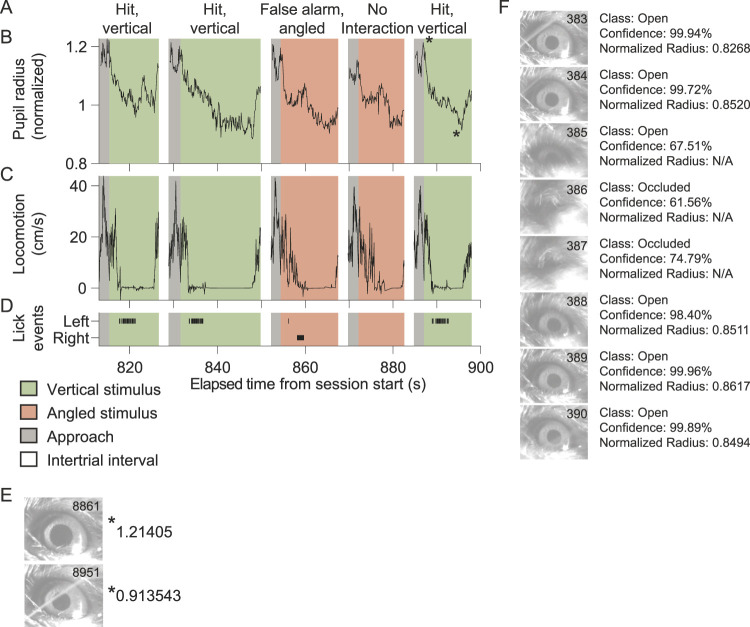
Integration and synchronization of data acquired from external devices with different sampling rates. ***A–D***, Synchronization of data acquired by external devices with lick event timestamps transmitted to the program from MCU #1. Trial outcomes (***A***), pupil radius normalized to the session-wide median radius (***B***), wheel travel velocity (***C***), and lick event timestamps for either the left or right side (***D***) during 5 consecutive trials in the head-fixed two-choice discrimination task for an example mouse. Data are aligned to time elapsed from the start of the session. ***E***, Normalized pupil radius is shown for the maximum and minimum values in the fifth trial shown in ***A***–***E***. Inset values depict frame numbers. ***F***, Analysis of pupil radius was performed on frames acquired from the eye tracking camera (external device), using a deep learning algorithm followed by a segmentation algorithm. Eight consecutive frames centered around a blinking occurrence from mouse shown in ***A–E***. Inset values depict frame numbers. The two-stage analysis approach facilitates the identification of ambiguous cases that are likely to warrant further user assessment. In the example shown, a partially occluded eye (frame #385) was classified as open, but the segmentation algorithm did not find a circle. Ambiguous frames can be displayed for the user to manually classify or alternatively adjust stringency of the segmentation algorithm.

Next, a custom MATLAB script, using the “imfindcircles” function, was used to segment the pupil. The radius and spatial position of the pupil was calculated for each frame containing an open eye. The radius was normalized to the median radius for the entire session (Fig. 5).

A known limitation of our pupillometry pipeline is that it is semiautomated, requiring user-assisted input. Currently, the classification of open versus occluded eye frames is accurate (100% test accuracy across 30 novel frames) and automated. However, in ∼1–3% of frames classified as having an open eye (and indeed, are confirmed to be fully open), the pupil fails to be segmented. This is currently addressed by having these frames displayed so that the user can manually click on the center of the pupil, specifically for the frames classified as open-eye and no pupil was segmented. Seeding the segmentation algorithm in this manner is sufficient for the pupil to be segmented. Future implementations could further automate this process.

#### Locomotion

In head-fixed preparations, upon session initiation, the encoder projects the current position in terms of the arbitrary encoder units at a rate of 300 Hz. If this position differs from the previous position projected, the MCU calculates the difference in encoder units and projects this difference to the master computer. This difference is recorded as well as the time the difference was registered, relative to epoch onset. In addition, the arbitrary encoder units are converted into centimeters, which determine the distance the subject has traveled. This distance is then converted to pixels which is then used to update the visual flow presented to the subject.

### Code accessibility

The code/software described in the paper is freely available online at https://github.com/Mdcrespo/BehavioralPlatform.

10.1523/ENEURO.0459-25.2026.d1 Data 1 Download Data 1, ZIP file.

10.1523/ENEURO.0459-25.2026.d2 Data 2 Download Data 2, DOCX file.

## Results

The behavioral platform described above was used to train mice in two distinct tasks, each with unique shaping protocols. Multiple microcontroller units (MCUs, e.g., Arduino Uno or Arduino Mega) were incorporated to couple external devices with the software program. At least one MCU is required to control the delivery of rewards (MCU #1; Fig. 1).

### Closed-loop two-choice discrimination task

To demonstrate the capabilities of the behavioral platform in a complex task, we implemented a closed-loop two-choice visual discrimination task, based on previous studies (Poort et al., 2015; Jeon et al., 2022; Poort et al., 2022). The visual stimulus presentation was coupled to the animal's locomotion (closed-loop) on a wheel (Movie 1). The stimulus presentation moved horizontally from the anterior to the posterior end of the monitor at a speed that was proportional to the rotation of the wheel. After running past a virtual approach wall composed of black and white circles overlaid on a gray background (“Epoch A”), mice were immediately presented with either a vertical (0°) or an angled (135°) grating stimulus (“Epoch B”), which drifted on the monitor in closed-loop format based on wheel speed. On vertical grating trials, mice were rewarded for licking left and on angled grating trials for licking right (Fig. 2*A*). In this implementation, the probability of either a vertical or angled trial was set to 0.50, with the stipulation that no more than three consecutive stimuli could be of the same type; this parameter can be changed in the script, offline. The isolation of each lick as a single decision is crucial when performing 2-choice tasks (Mayrhofer et al., 2013; Goltstein et al., 2018). Because of this need to isolate licking, the custom-made lick-port assembly was designed with a divider positioned in between the two lick-ports (Fig. 2*B*). The purpose of this divider was to prevent simultaneous tongue contact to both lick-ports (Fig. 2*B*). We noted that the tongue trajectory followed an upward sweeping motion. Therefore, the divider was designed to have added downward height, the purpose of which is such that the divider reached below the lick-ports when mounted in front of the animal's mouth (Extended Data Fig. 2-1*A*). The divider was designed in such a way that the distance between the end of the divider relative to the end of the lick-ports could be manually adjusted to best orient in relation to the mouse performing the task (Extended Data Fig. 2-1*B*). Our task design allowed two different behavioral discrimination assessments to be made: Epoch A versus Epoch B (the approach epoch was not rewarded) and grating orientation discrimination within Epoch B (Fig. 2*C*). Generally, subjects first exhibited licking in both epochs of the task (Fig. 2*D*, top). During the initial stages of training, licking was restricted to the grating epoch (Fig. 2*D*, middle), indicating mice learned not to lick during the approach epoch. With further training, discrimination of trial type (e.g., grating angle discrimination, vertical vs angled) emerged. For trials in which the left port was rewarded (vertical stimulus), the number of lick events at the right port (nonrewarded) decreased, and similarly, for trials in which the right port was rewarded (angled stimulus), the number of lick events at the left port (nonrewarded) decreased (Fig. 2*D*). In summary, as mice gained more experience, lick events were restricted to the specific time windows and conditions that resulted in reward delivery.

10.1523/ENEURO.0459-25.2026.f1 Figure 2-1 Illustration of the head-fixed behavioral station, top-down view. Distances (black lines of the visual stimulus screen and pupil recording camera relative to the mouse. The center of the pupil camera and screen were aligned to the height of the eyes. IR light source can be moved to best illuminate the pupil; as performed, IR light was 5 cm lower than the mouse and angled up 10° relative to horizontal to illuminate the pupil (this angle not shown). Lick-port is shown as a white square, suspended over the wheel (blue) by a rod (gray, horizontal line). Head-fixed mouse is held in position by a bar (gray, vertical line). Dashed gray line indicates the midline of the mouse. Arrows indicate direction of IR illumination. Download Figure 2-1, TIF file.

10.1523/ENEURO.0459-25.2026.f2 Figure 2-2 Dimensions of the 2 lick-port holder and divider. **A)** Dimensions from the side view (left) and isometric view (right) of the 2 lick-port holder and divider in relation to two water tubes (gray). The front of the divider (water delivery side) features an added downward height. This feature creates a barrier such that for each lick trajectory (which is characterized as an upward motion, starting near the lower jaw) is isolated to one side. Connection location for the plastic tubing as well as the capacitive sensor to the metal water tube shown (left). **B)** Dimensions from the top view (left) and front view (right) of the 2 lick-port holder and divider in relation to two water tubes (gray). The distance that the divider protrudes from the water tubes on the water delivery side can be adjusted as needed. All dimensions are reported in mm. Download Figure 2-2. Dimensions of the 2 lick-port holder and divider., TIF file.

To determine whether the trend was present in all six animals, we identified the first session number in which the *d*-prime value was ≥1 for both Epoch A versus Epoch B discrimination (epoch discrimination_dp1_) and grating orientation discrimination (grating discrimination_dp1_). A *d*-prime of 1 was selected as the threshold for this calculation because a value of 1 means that the two distributions being compared are 1 standard deviation apart. The median difference between grating discrimination_dp1_ − epoch discrimination_dp1_ was 7.5 sessions. The difference was statistically significant [one-sample Kolmogorov–Smirnov test (sample size = 6), *p* = 2.77 × 10^−10^]. To confirm the effect was robust to the choice of threshold, we performed the same calculation except the threshold was set to a *d*-prime of 1.5. In this case the one-sample Kolmogorov–Smirnov test *p* value was 1.11 × 10^−16^.

Prior to training in the discrimination task, mice were subjected to 1–2 d of shaping. Shaping in this case involved training the mice to walk for a water reward. Out of seven mice with successful surgeries, all seven were subjected to shaping. One out of six mice did not walk consistently on the wheel and was removed from the experiment. All remaining six mice were trained and all six learned the closed-loop task (Fig. 2*E*), achieving a performance *d*-prime ≥1.5 for at least 3 consecutive days.

One challenge often faced when training mice in two-choice tasks is the expression of bias toward one specific lick-port (Marbach and Zador, 2017; Goltstein et al., 2018; Bari et al., 2019; Aguillon-Rodriguez et al., 2021; Do et al., 2023). Indeed, five out of the six mice developed a bias toward either the left or right lick-port. Two mice developed a bias to the left and three to the right, indicating the bias was not due to hardware differences between the two ports. Bias was operationally defined as an absolute side-bias index >1. To overcome side-bias, reward size was increased on the nonpreferred side, and once the bias was no longer observed, reward size was equalized for each side. The example mouse shown in Figure 3 demonstrates that this approach can successfully remove bias. The bias was progressively decreased during the sessions in which extra water was given, and once the reward size was re-equalized between the two sides, a bias did not re-emerge. This trend was observed in all five mice that had a bias.

### Freely moving luminance discrimination task

To demonstrate the design flexibility of the behavioral platform, we implemented a freely moving operant conditioning visual discrimination task (Fig. 4*A*). The goal of the task was for mice to discriminate between a white or black set of circles, presented on a gray background (Fig. 4*B*). The choice of stimulus presentation is flexible; in addition to luminance discrimination, acuity or detection tasks, as well as cortical-dependent tasks, can be implemented using this platform. Within 3–5 sessions, mice were able to maintain a low false alarm rate of 20% or less.

One wild-type and one haploid mutant mouse were trained in the freely moving luminance discrimination task, after undergoing two stages of shaping. Generally, in the first session mice licked indiscriminately regardless of which stimulus was presented, resulting in low performance (calculated as *d*-prime) and a high hit rate. As the session progressed in the example mouse shown in Figure 4*C* and *D*, the false alarm rate decreased within the first session. In the last 100 trials of the session, licking was largely restricted to the rewarded stimulus presentation (Fig. 4*C*,*D*). After several training sessions, the false alarm rate was maintained below 20% for the session duration (Fig. 4*E*).

Freely moving behavioral paradigms are useful for high-throughput training or in which more complex behavioral outputs are desired. Here we demonstrated that mice harboring a mutation associated with autism, *Shank3-*Δ*c*, can also learn this task. Similar to the wild-type mouse, the mutant mouse maintained a low false alarm rate after several training sessions (Fig. 4*F*).

In paradigms in which subjects consistently achieve a high hit rate, false alarm rate drives the *d*-prime score. This relationship can easily be seen in both the wild-type and the mutant examples. Note, in Figure 4*F* the false alarm rate is close to zero for a subset of trials (approximately trial numbers 200–250). This decrease in false alarm rate is associated with an increase in the *d*-prime value. It is unclear what drives a subject to fluctuate between a false alarm rate of 0–15%. Although we did not incorporate a punishment or deterrent, such as an air-puff or time-out, adding a deterrent to licking on No-go trials will often reduce such fluctuations. It is possible to incorporate punishments or deterrents in the program.

### Synchronization of external devices

Next, using the closed-loop two-choice task, we demonstrate that trial outcomes, data acquired from external devices such as pupil radius and locomotion, and lick events can be easily aligned to epochs within trials (Movie 1). These elements are visualized by aligning data to the elapsed time from the start of the session (Fig. 5*A*–*D*). Individual data items acquired from an external device can be easily referenced, such as specific frames from the eye tracking camera (Fig. 5*E*).

Eye tracking, including pupillometry, is an integral part of most head-fixed perceptual paradigms. In this example we show the utility of our two-stage process for analyzing pupil radius. To extract pupil metrics, the ResNet18 model was used to first classify eyes as open or occluded (eye lids closed). The presented class as well as the class confidence was stored for each frame. Next, the pupil was segmented in frames classified as open (Fig. 5*F*). Frames can be scored as ambiguous in the case the eye is classified as open, but the pupil fails to be segmented. This can happen if the eye is partially closed. These ambiguous frames can be curated and brought to the attention of the user for further consideration.

## Discussion

As rodent behavioral task designs become increasingly complex, there is a growing need for affordable platforms that can be used in a variety of scenarios (Kato et al., 2015; Berditchevskaia et al., 2016; Jurjut et al., 2017). To assist in this need, we introduce a low-cost behavioral platform well-suited for flexibly implementing a variety of behavioral task designs. A standardized trial-by-trial data organization facilitates rapid analysis of behavior and the scoring of trial outcomes. As such, there is a quick turnaround between running experiments and visualizing the behavioral results. Similarly, it is straightforward for end-users to acquire and analyze data from different task designs. The behavioral platform is built from inexpensive hardware and allows for the creation of both head-fixed as well as freely moving paradigms. As proof of concept, two distinct tasks were presented here, a head-fixed closed-loop two-choice discrimination task, as well as a freely moving operant conditioning task. Our platform addresses the need for flexibility in design as head-fixed tasks become increasingly complex and require sophisticated shaping protocols (Guo et al., 2014; Fiser et al., 2016; Henschke et al., 2020) and is suitable for use in both head-fixed preparations as well as cases in which head-fixation may introduce confounds or higher-throughput is desired, such as when studying the impact of genetic mutations (Atlan et al., 2018; Kim et al., 2019; Juczewski et al., 2020; Yan, 2024).

Complex tasks often require fine-tuning of task parameters to optimize training during behavior (Kato et al., 2015; Berditchevskaia et al., 2016; Bari et al., 2019). The behavioral platform presented offers two solutions for this process. First, a live readout of behavioral performance throughout the task allows the researcher to have day-of analysis of performance, allowing the best optimization of task criteria to ensure quick adoption of desired behavioral responses by the subject. Second, within-session modulation of task parameters, such as automatic water delivery on missed trials to maintain engagement or reducing side-bias in the case a two lick-port reward delivery system is used. While these fine-tuning abilities allow for direct alterations of parameters at the researcher's discretion, a future direction could be the automation of these alterations if the researcher is unable to monitor subjects or there is a need to increase throughput.

Not only are behavioral tasks becoming increasingly complex, but the number of associated physiological and/or motor-related events simultaneously captured is also increasing. Our platform is modular in design and allows users to add additional external devices without changing the fundamental structure of the data acquisition system. This facilitates testing and troubleshooting the integration of a new device into existing systems. Indeed, the ease with which users can identify timestamped data frames is demonstrated in Figure 5.

In our system, as well as any water-reward delivery system, the upper limit on the number of trials performed is set by the daily volume of water rationed relative to the volume of each individual reward. In the head-fixed example we provided, the daily water ration was 750 µl, and the drop size was 6 µl. Therefore, considering every trial could be rewarded, the upper limit on the number of successful trials was 125. In the freely moving paradigm, the daily water ration was 750 µl, and the drop size was 4 µl. In addition, only 30% of the trials were rewarded. Therefore, the upper limit on the number of successful trials was 635. It is possible to increase the number of expected trials by increasing the daily ration of water or decreasing the drop size (Cruz et al., 2025; Zhong et al., 2025). Another known limit of our system is the use of a capacitive sensor in the lick-port. Capacitive sensors are likely to generate electrical artifacts in electrophysiological recordings. This limitation can be overcome by replacing capacitive sensors with optical sensors in which tongue deflections are detected by breaks in a photodiode beam (Williams et al., 2018; Jeon et al., 2022; Silva et al., 2024). Given optical sensors can generate TTL outputs these devices can be coupled to Arduino microcontrollers, and as such they can be integrated into our system for use in electrophysiology experiments.

In summary, the behavioral platform presented allows for the creation of a wide range of behavioral tasks and stimulation of different sensory modalities. The output is organized in a standardized, trial-by-trial format capable of synchronizing many external devices for both subject interaction and recording, thereby providing the opportunity to easily test multiple novel hypotheses with specified tasks optimized for each of the hypotheses.

## Synthesis

Reviewing Editor: Michael McDannald, Boston College Decisions are customarily a result of the Reviewing Editor and the peer reviewers coming together and discussing their recommendations until a consensus is reached. When revisions are invited, a fact-based synthesis statement explaining their decision and outlining what is needed to prepare a revision will be listed below. The following reviewer(s) agreed to reveal their identity: NONE.

Synthesis:

This manuscript details an effective low cost system for training mice in a variety of experimental settings. Convincing data are shown for visual discrimination. Further, sufficient details are given for implementation by other laboratories. The reviewer and the reviewing editor see the value in this manuscript. Below are comments by the reviewer designed to strengthen the manuscript. Please address each.

Reviewer Comments:

Please see below some comments/suggestions:

Introduction:

• When mentioning "Behavioral tasks performed by head-fixed animals are used when study designs require precise and repeated longitudinal monitoring...", It is worth noting that another field where head-fixed experimentation are very important is awake fMRI. You can add references for examples of recent open-source design for awake fMRI head-fixation setups.

• In general, experiments in freely moving animals are preferred and head-fixed setups are used when they are required due to some reasons. The way the introduction is set up, does not suggest this. It is worth having this reminded in the introduction.

• You mention that your solution is compatible with 'neurophysiological imaging'. Is it compatible with any sort of electrophysiological recording (maybe for LFP recording sue to the sample rate limitation)? If so, it is worth mentioning it.

Materials and Methods:

• Add an image or drawing of the head-fix behavioral platform. You can put it in a supplementary figure as it is not part of the paper. But it helps the reader to understand what is going on better.

• You mention "a detailed protocol of hardware assembly and software installation, all code used, as well as relevant information for implementation are available freely", but a detailed protocol of hardware assembly is not provided.

• Is it possible to increase the sampling rate above 300Hz? For example, to include electrophysiological (silicone probe, etc.) data?

• When mentioning "During water restriction, mice were given 750μL per day.", specify for how many days water restriction was applied?

• When mentioning "Lick-ports were built from a 0.094-inch-outer diameter stainless steel water tube with a 0.02-inch-wall thickness (6100K441, McMaster-Carr) connected to a capacitive sensor (SparkFun, AT42QT1011)", I suggest that for clarity, you highlight the sensor location in the related figure.

• In 'Acclimation to Task Environment', please clarify whether the mice were water deprived before this and include the related details?

• In 'Two-choice Discrimination Task', - correct the typo: 'diameter (radius?)'.

• In 'Two-choice Discrimination Task', when you mention 'or after 90

• minutes passed since session onset.', clarify if mice did not collect enough water, were they given the remaining amount?

• In 'Acclimation to Task Environment, please clarify whether the mice were water deprived before this and include the related details?

• When mentioning "begin. Once the mouse was able to collect the daily water allotment within a 90-minute session, the mouse was transitioned into a second shaping task. The second shaping task follows the initial task with the addition of self-initiation. Within the task, the mouse must touch the trial-initiation sensor to trigger stimulus presentation. Upon stimulus presentation, if the mouse licked the lick-port, a water reward was released. 0.5 seconds after reward delivery stimulus would disappear, and the next trial would begin after a 0.5 second inter-trial interval. Once the mouse was able to collect the daily allotment of water within a 90-minute session, the mouse was moved into the luminance discrimination task.", in this experimental design, the mouse could have more than daily water allowance. Wouldn't this affect the performance of the mouse during the second task?

• Details of 'luminance discrimination task' is not described in the method

• In 'Freely Moving Behavior Task', when mentioning 'A brief inter-trial-interval is required...', how long is that?

• In 'Pupillometry', it would be useful to share what camera was used.

• In 'Locomotion', mention that this is for headfixed mice.

Results:

• In 'Closed-Loop Two-Choice Discrimination Task', correct the typo: '(From preliminary video recordings?)'.

• When mentioning 'Therefore, the divider was designed to have added downward height, the purpose of which is such that the divider reached below the lick-ports when mounted in front of the animal's mouth (Fig. 2-1a).', this point is not clear in the figure. add a note etc. to make it clear there.

• When mentioning 'With further training, mice learned to discriminate grating orientation (Fig. 2D).', add more details to explain results from figure 2D.

• When mentioning 'Prior to training in the discrimination task, mice were subjected to 1-2 days of shaping', clarify if mice were water deprived before.

• When noting 'and once the reward size was re-equalized between the two sides, a bias did not reemerge.', please clarify if this was the case for all the animals concerned with bias.

• When mentioning 'After several training sessions, the false alarm rate was maintained below 20% for the session duration (Fig. 4E).', provide an estimate average number of training sessions.

• In Fig. 4F, is there an explanation why performance first increases then decreased?

Discussion:

• You noted that 'While these fine-tuning abilities allow for direct alterations of parameters at the researcher's discretion, a future direction could be the automation of these alterations if the researcher is unable to monitor subjects or there is a need to increase throughput.'. In the method and result sections, it was not clear that the modification is made by the observer and is not automatic. Please make this clear there.

• Correct the sentence in '(and are indeed, are confirmed to be fully open),'

• In discussion, briefly explain what is the limit of trial numbers based on the reward volume.

Figure 2:

• B: For more clarity, put a real 3d sketch. The tubes do not look 3d.

• C: add statistical test for comparison between early and later sessions.

## Author Response

We sincerely appreciate the constructive and thoughtful comments provided by the reviewers. The manuscript is improved. We addressed all concerns below. In addition, we moved one paragraph out of the Discussion to the Methods, Pupillometry section. We also added a statement in the Discussion regarding the limits of using capacitive sensors in electrophysiology experiments and describe an alternative that is compatible with our system. Insertions are highlighted in gray, in the 'MarkUp' copy.

Synthesis of Reviews:

Computational Neuroscience Model Code Accessibility Comments for Author (Required):

Authors appear to be compliant.

Synthesis Statement for Author (Required):

Synthesis:

This manuscript details an effective low cost system for training mice in a variety of experimental settings. Convincing data are shown for visual discrimination. Further, sufficient details are given for implementation by other laboratories. The reviewer and the reviewing editor see the value in this manuscript. Below are comments by the reviewer designed to strengthen the manuscript. Please address each.

Reviewer Comments:

Please see below some comments/suggestions: Introduction: • When mentioning "Behavioral tasks performed by head-fixed animals are used when study designs require precise and repeated longitudinal monitoring...", It is worth noting that another field where head-fixed experimentation are very important is awake fMRI. You can add references for examples of recent open-source design for awake fMRI head-fixation setups.

Thank you for this suggestion; added as recommended, line 55. • In general, experiments in freely moving animals are preferred and head-fixed setups are used when they are required due to some reasons. The way the introduction is set up, does not suggest this. It is worth having this reminded in the introduction.

We updated this section as suggested, starting on line 44. • You mention that your solution is compatible with 'neurophysiological imaging'. Is it compatible with any sort of electrophysiological recording (maybe for LFP recording sue to the sample rate limitation)? If so, it is worth mentioning it.

Yes, our solution is compatible with 'neurophysiological imaging' and electrophysiology (silicone probe, etc.) data, thank you for raising this issue, it is important that this is clear to the readers. This is clarified, line 110. Materials and Methods: • Add an image or drawing of the head-fix behavioral platform. You can put it in a supplementary figure as it is not part of the paper. But it helps the reader to understand what is going on better.

Thank you for this suggestion. Updated as requested, we added Supplementary Figure 2-1. • You mention "a detailed protocol of hardware assembly and software installation, all code used, as well as relevant information for implementation are available freely", but a detailed protocol of hardware assembly is not provided.

The protocol for hardware assembly was added, and will be available in the GitHub directory. The file for review is named 'HardwareProtocol'. • Is it possible to increase the sampling rate above 300Hz? For example, to include electrophysiological (silicone probe, etc.) data? We addressed this comment in the Behavioral Platform section of the Methods, starting at line 110. • When mentioning "During water restriction, mice were given 750μL per day.", specify for how many days water restriction was applied? The procedure is now clarified, starting on line 156. Length of restriction is clarified on line 161. • When mentioning "Lick-ports were built from a 0.094-inch-outer diameter stainless steel water tube with a 0.02-inch-wall thickness (6100K441, McMaster-Carr) connected to a capacitive sensor (SparkFun, AT42QT1011)", I suggest that for clarity, you highlight the sensor location in the related figure.

The sensor location is now indicated in Supplementary Figure 2-2 and clarified on line 183. • In 'Acclimation to Task Environment', please clarify whether the mice were water deprived before this and include the related details? This issue should now be clear, given updates starting at line 156. • In 'Two-choice Discrimination Task', - correct the typo: 'diameter (radius?)'.

Thank you, we confirmed diameter is correct and the typo is now fixed. • In 'Two-choice Discrimination Task', when you mention 'or after 90 minutes passed since session onset.', clarify if mice did not collect enough water, were they given the remaining amount? Yes, mice were always supplemented in the case they did not earn their daily ration during a session. We clarified this on line 162. • In 'Acclimation to Task Environment, please clarify whether the mice were water deprived before this and include the related details? This is now clarified in the Animal Housing and Preparation section, starting on line 156. • When mentioning "begin. Once the mouse was able to collect the daily water allotment within a 90-minute session, the mouse was transitioned into a second shaping task. The second shaping task follows the initial task with the addition of self-initiation. Within the task, the mouse must touch the trial-initiation sensor to trigger stimulus presentation. Upon stimulus presentation, if the mouse licked the lick-port, a water reward was released. 0.5 seconds after reward delivery stimulus would disappear, and the next trial would begin after a 0.5 second inter-trial interval. Once the mouse was able to collect the daily allotment of water within a 90-minute session, the mouse was moved into the luminance discrimination task.", in this experimental design, the mouse could have more than daily water allowance. Wouldn't this affect the performance of the mouse during the second task? Mice were moved onto the discrimination task on the next day, not the same day. Thank you for catching this. This is now clarified, starting on line 328. • Details of 'luminance discrimination task' is not described in the method Details are added, starting on line 337. • In 'Freely Moving Behavior Task', when mentioning 'A brief inter-trial-interval is required...', how long is that? The minimum interval required is 50 ms, in the example case we set this interval to 500 ms. This information was added, line 348. • In 'Pupillometry', it would be useful to share what camera was used.

Updated as suggested on line 371. • In 'Locomotion', mention that this is for headfixed mice.

Updated as suggested, line 410. Results: • In 'Closed-Loop Two-Choice Discrimination Task', correct the typo: '(From preliminary video recordings?)'.

Typo corrected. • When mentioning 'Therefore, the divider was designed to have added downward height, the purpose of which is such that the divider reached below the lick-ports when mounted in front of the animal's mouth (Fig. 2-1a).', this point is not clear in the figure. add a note etc. to make it clear there.

The figure was updated to make this clearer. • When mentioning 'With further training, mice learned to discriminate grating orientation (Fig. 2D).', add more details to explain results from figure 2D.

Updated as recommended, starting at line 447. • When mentioning 'Prior to training in the discrimination task, mice were subjected to 1-2 days of shaping', clarify if mice were water deprived before.

Yes, mice were water deprived before. This should now be clear, given the updates starting at line 156. • When noting 'and once the reward size was re-equalized between the two sides, a bias did not reemerge.', please clarify if this was the case for all the animals concerned with bias.

Thank you for this comment. We clarified that all mice with a bias were corrected, and that a bias did not reemerge, line 472. • When mentioning 'After several training sessions, the false alarm rate was maintained below 20% for the session duration (Fig. 4E).', provide an estimate average number of training sessions.

Within 3-5, this is now updated, line 481. • In Fig. 4F, is there an explanation why performance first increases then decreased? An explanation is provided starting on line 494, as well as a suggestion for more tightly regulating the false alarm rate. Discussion: • You noted that 'While these fine-tuning abilities allow for direct alterations of parameters at the researcher's discretion, a future direction could be the automation of these alterations if the researcher is unable to monitor subjects or there is a need to increase throughput.'. In the method and result sections, it was not clear that the modification is made by the observer and is not automatic. Please make this clear there.

This was made clear, line 224, 257, 260. In addition, triggering of one of the features does not require observer input, we clarified on line 232. In addition, we provide a new chart (Table 2) highlighting the parameters that can be changed by the observer during a session. • Correct the sentence in '(and are indeed, are confirmed to be fully open),' Corrected. • In discussion, briefly explain what is the limit of trial numbers based on the reward volume.

Updated as recommended, starting on line 551. Figure 2: • B: For more clarity, put a real 3d sketch. The tubes do not look 3d.

Updated as requested. • C: add statistical test for comparison between early and later sessions.

A one-sample Kolmogorov-Smirnov (K-S) test was performed using all animals, results are reported starting on line 449. Details: In all 6 mice the first session in which the d-prime value was {greater than or equal to} 1 for corridor discrimination occurred at least two days before the d-prime value was {greater than or equal to} 1 for grating discrimination. In other words, the difference in number of sessions to cross a d-prime threshold of 1 was at least two sessions. The median difference (e.g. the number of sessions that corridor discrimination crossed a d-prime threshold of 1 prior to grating discrimination across the 6 mice) was 7.5 sessions. To determine whether the effect was significant, we asked whether the difference distribution was skewed. The null hypothesis was that the distribution was normal (e.g. the median difference was 0).
