# Supplementary material for: Open-Source Platform for Adjustable Training Regimes in Freely Moving and Head-Fixed Mice
Source: eNeuro. 2026 Mar 10;13(3):ENEURO.0459-25.2026. doi: 10.1523/ENEURO.0459-25.2026 (PMC13045870; doi:10.1523/ENEURO.0459-25.2026)
Supplement: Data 1 — Download Data 1, ZIP file. [file eneuro-13-ENEURO.0459-25.2026-s001.zip › ExtendedData2/PartsFiles/PartsList.docx]

Parts List:

2Choice Task:

Capacitive touch sensor X2 - (SparkFun, AT42QT1011)

Metal lick spout X2 – (6100K441, McMaster-Carr)

Plastic tubing – (R-3603, Tygon) – discontinued and replaced by E-3603 which is same but does not use DEHP

- Wheel – Foam Roller- ER-EVA-3002BD, <https://www.amazon.com/gp/product/B0BGJ1WTV9/ref=ppx_yo_dt_b_asin_title_o04_s01?ie=UTF8&psc=1> OR (<https://www.amazon.com/Living-Fit-Foam-Roller-Length-Diameter/dp/B0BR65JNYM/ref=sr_1_97?hvadid=616863318906&hvdev=c&hvlocphy=9005925&hvnetw=g&hvqmt=e&hvrand=7799337739690832263&hvtargid=kwd-320901136&hydadcr=24664_13611849&keywords=body%2Broller&qid=1683053860&sr=8-97&th=1>)
- Shaft – McMaster-Carr Rotary Shaft, ¼” diameter, carbon steel, 12” long (part number: 1327K66) (<https://www.mcmaster.com/products/rotary-shafts/rotary-shafts-5/diameter~1-4/>)
- Ball Bearings R4-2Z (60355K43). <https://www.mcmaster.com/products/bearings/ball-bearings-8/for-shaft-diameter~1-4/>
- Rotary encoder – US Digital, part **E5-1250-250-IE-S-H-R-3**. E5 Options: 1250 cpr (counts per revolution), ¼” bore size, index, Single-Ended output, R-base and H-cover, R base = 1.812" Diameter Bolt Circle, 3 Slot Rotational Mounting (R), H-cover = Through-hole cover (H)). We are not sure whether need indexed or not… to be safe, currently getting index

<https://www.usdigital.com/products/encoders/incremental/kit/e5/>

- Cable- US Digital, part **CA-FC5-W4-NC-x**, where x is length of cable in feet**.** <https://www.usdigital.com/products/accessories/cables/5-pin/ca-fc5-w4-nc/?q=CA-FC5-W4-NC-3>
- Thor labs parts: 1x 8”-by-8” plate, 2x TR10, 1x TR3, 3x RA90, 2x PH2

Order info, Pin connectors:

<https://www.mouser.com/ProductDetail/TE-Connectivity/826629-3?qs=AplfTeSvkkA3hKOe%252BWTzDA%3D%3D&mgh=1&gclid=CjwKCAjw-vmkBhBMEiwAlrMeFwyVEUrb9Ne9HQ1WTpxpnMHWprsI9DKh15DWFaAJKUo1o8Nz38VQxRoCs8sQAvD_BwE>

Order info, Jumper wires:

<https://www.mouser.com/ProductDetail/SparkFun/PRT-12796?qs=WyAARYrbSnYazPSZHWXCfQ%3D%3D&mgh=1&gclid=CjwKCAjw-vmkBhBMEiwAlrMeF9DtKZt6mAqAAFwN53jBEEPZh9R822uc0H9eH7Ck8v_rgOIdPDmPNBoCPToQAvD_BwE>

Monitor – (Dell; 30”, 2560×1600 resolution)

Solenoid x2 – (Lee Company; LHDA1233115H)

Water spout holder – Custom 3d printed

Relay x2 – 4411-TS0010D-ND, Digikey

Camera – (Teledyne FLIR, FL3-U3-13E4M-C)

IR light source – (850 nm)

Arduino uno x2 – (A000066, Arduino)

Arduino mega - (A000067, Arduino)

Free Behavior Task:

Capacitive touch sensor X2 - (SparkFun, AT42QT1011)

Metal lick spout – (6100K451, McMaster-Carr)

Metal initiation rod – (6100K451, McMaster-Carr)

Plastic tubing – (R-3603, Tygon)

Monitor – (Dell; 20”, 1600x900 resolution)

Solenoid – (Lee Company; LHDA1233115H)

Relay – 4411-TS0010D-ND, Digikey

Arduino mega - (A000067, Arduino)
