# Supplementary material for: Open-Source Platform for Adjustable Training Regimes in Freely Moving and Head-Fixed Mice
Source: eNeuro. 2026 Mar 10;13(3):ENEURO.0459-25.2026. doi: 10.1523/ENEURO.0459-25.2026 (PMC13045870; doi:10.1523/ENEURO.0459-25.2026)
Supplement: Data 2 — Download Data 2, DOCX file. [file eneuro-13-ENEURO.0459-25.2026-s005.docx]

Protocol for behavioral platform

Table of Contents

[I. Closed-loop 2-choice visual discrimination task: 2](#_Toc219815136)

[MCU # 1 – Lick-port controller 3](#_Toc219815137)

[MCU # 2 – Locomotion recording 5](#_Toc219815138)

[MCU # 3 – Pupil tracking and uncoupled relay 7](#_Toc219815139)

[II. Freely moving luminance discrimination task: 8](#_Toc219815140)

[MCU # 1 – Lick-port controller 9](#_Toc219815141)

[III. User Notes: 11](#_Toc219815142)

[IV. Parts List 12](#_Toc219815143)

# Closed-loop 2-choice visual discrimination task:


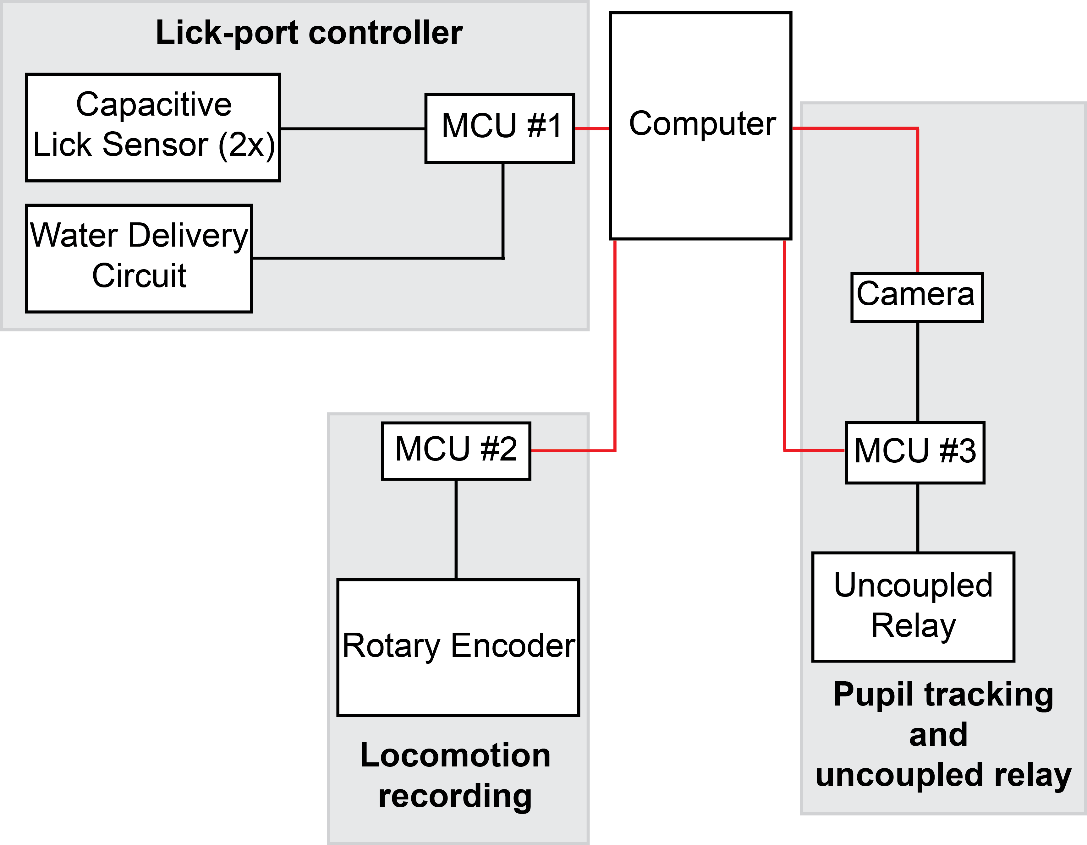


**Overview of components:**

Overview of the connections between the computer, the 3 MCU used in the closed-loop 2-choice visual discrimination task, the lick-port components including the capacitive lick sensors for lick detection and the water delivery circuit for reward administration, as well as 3 external devices, the uncoupled relay, the rotary encoder, and the camera. Connections in red indicate communication between components via USB, while connections in black indication communication between components via digital I/O signals.

Each MCU is connected to the computer and individual components of the platform utilized during the closed-loop 2-choice visual discrimination task. Below are detailed wiring diagrams for each MCU and the components which communicate with that specific MCU.

**Important Note:** Wires are color coded between components and labeled such that each of the two labels for a given wire are where the given wire is supposed to be connected on each end. For example, in MCU #1, the yellow wires indicate connections between MCU #1 and the relay module, while the wire labelled “Water Valve Control Power” specifically indicates a wire should be connected on one side to MCU #1 pin 11 and on the other side the VCC pin on the relay module.

## MCU # 1 – Lick-port controller

**
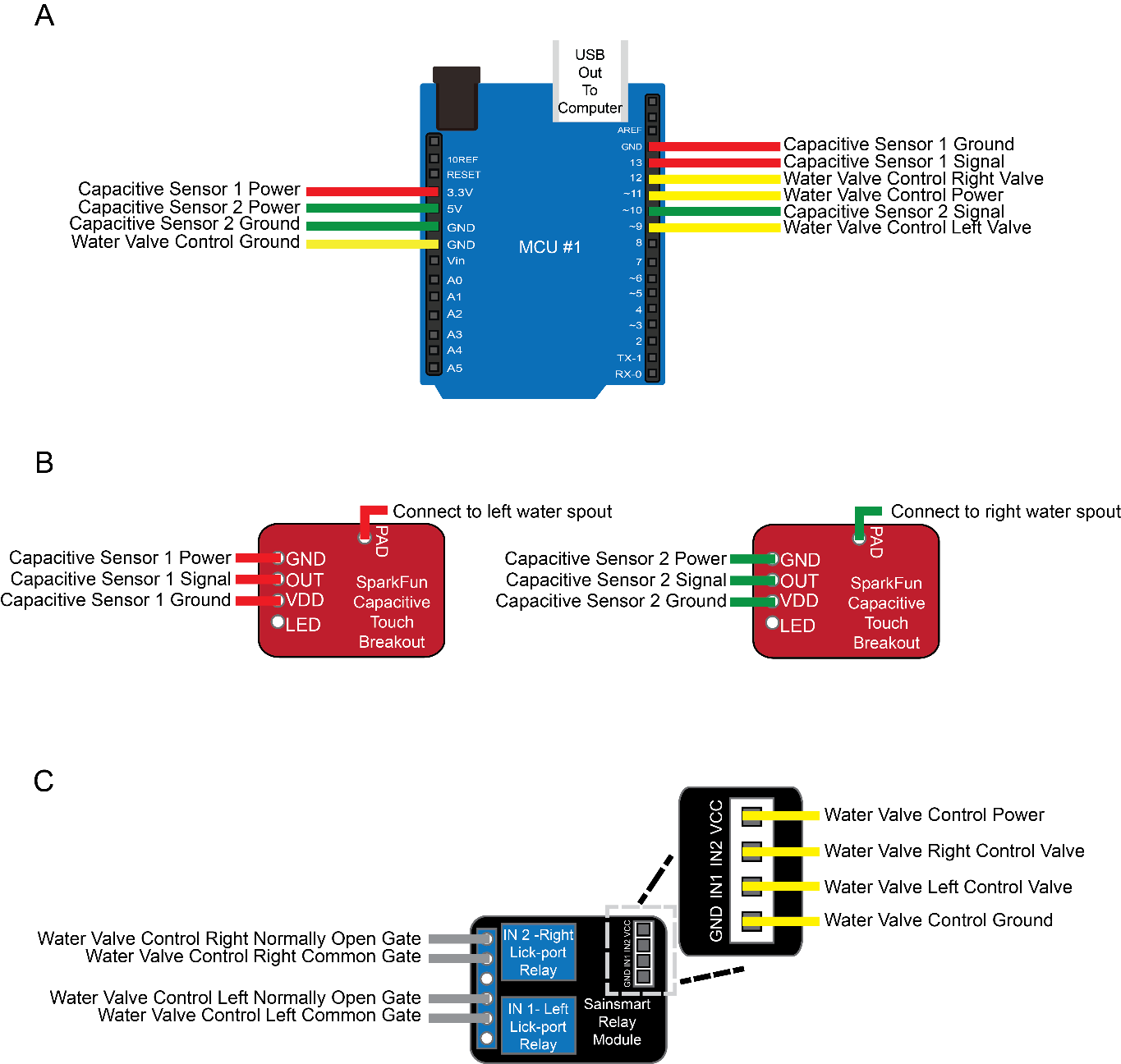
**

**Wiring diagram for MCU #1, capacitive touch breakouts, and relay module:**

1. Wires connected to MCU #1. MCU # 1 is connected to 3 components of the platform. Two Sparkfun capacitive touch breakouts, shown in red wire for breakout 1 and green wire for breakout 2, and the Sainsmart relay module, shown in yellow wire.
2. Wires connected to Sparkfun capacitive sensors. Each sensor receives 3 wires from MCU #1, a wire for powering the component, a wire for grounding the component, and a wire for outputting touch detections to MCU #1. In addition, each breakout has a wire connected to the PAD section, which is directly connected to the water spout, to allow the breakout to detect touches of the water spout. Capacitive sensor 1 should be connected to the left water spout and capacitive sensor 2 should be connected to the right water spout.
3. Wires connected to Sainsmart relay module. The Sainsmart relay module contains relays for both the left and right lick-port (left). Two wires, shown in gray, are connected to each relay in using the upper two pins for the relay. This causes the relay to be in open state normally, preventing water flow unless when triggered. Triggering is caused by 4 wires from MCU #1 connected to the module, shown in yellow (right). From MCU #1 there is a wire to power the module, a wire to ground the module, a wire to trigger reward delivery through the left lick-port, and a wire to trigger reward delivery through the right lick-port. The relay wires are connected to the water delivery circuit to cause this reward delivery, which is detailed below.


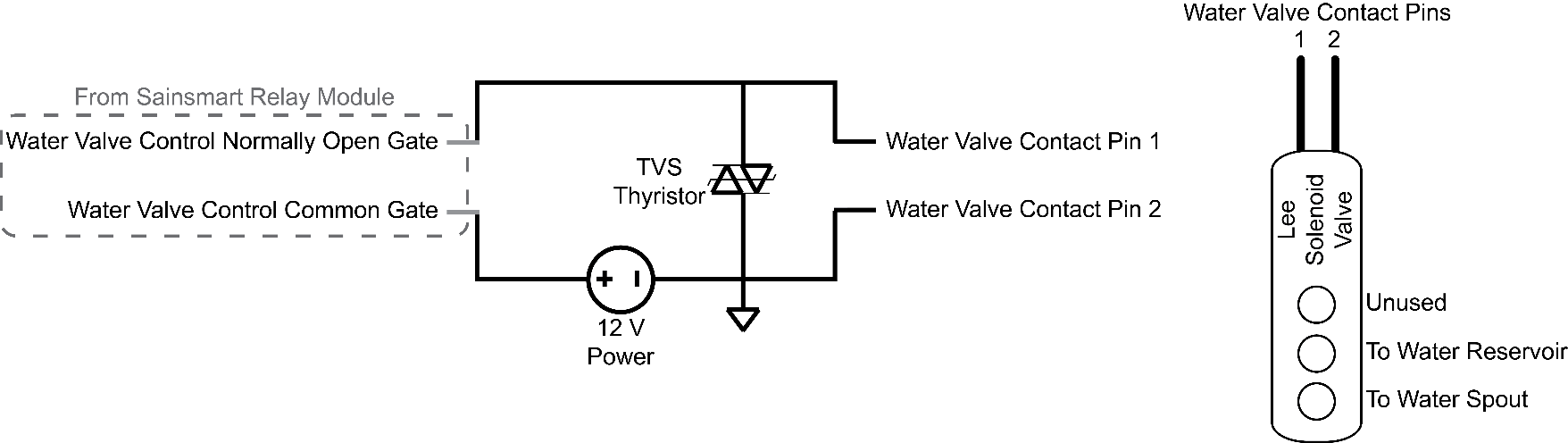


**Circuit diagram for water delivery circuit:**

A water delivery circuit is created for each lick-port (left). For the Closed-loop 2-Choice visual discrimination task two water delivery circuits will be created. The water delivery circuit consists of 4 elements: the Sainsmart relay module, responsible for gating water delivery timing, the Lee solenoid valve, responsible for delivering water from reservoir to the metal water spout, the 12 V power supply, responsible for powering the solenoid, and the TVS thyristor, to prevent inductive kickback and protect against power surges. The Lee solenoid valve (right) is connected to the other components of the water delivery circuit via two contact pins located on the top of the valve. To facilitate water delivery, the water reservoir is connected to the center valve port, and the rubber tubing connected to the back of the lick-port is connected to the bottom valve port. This ensures that water flow is prevented, unless reward delivery is signaled.

## MCU # 2 – Locomotion recording


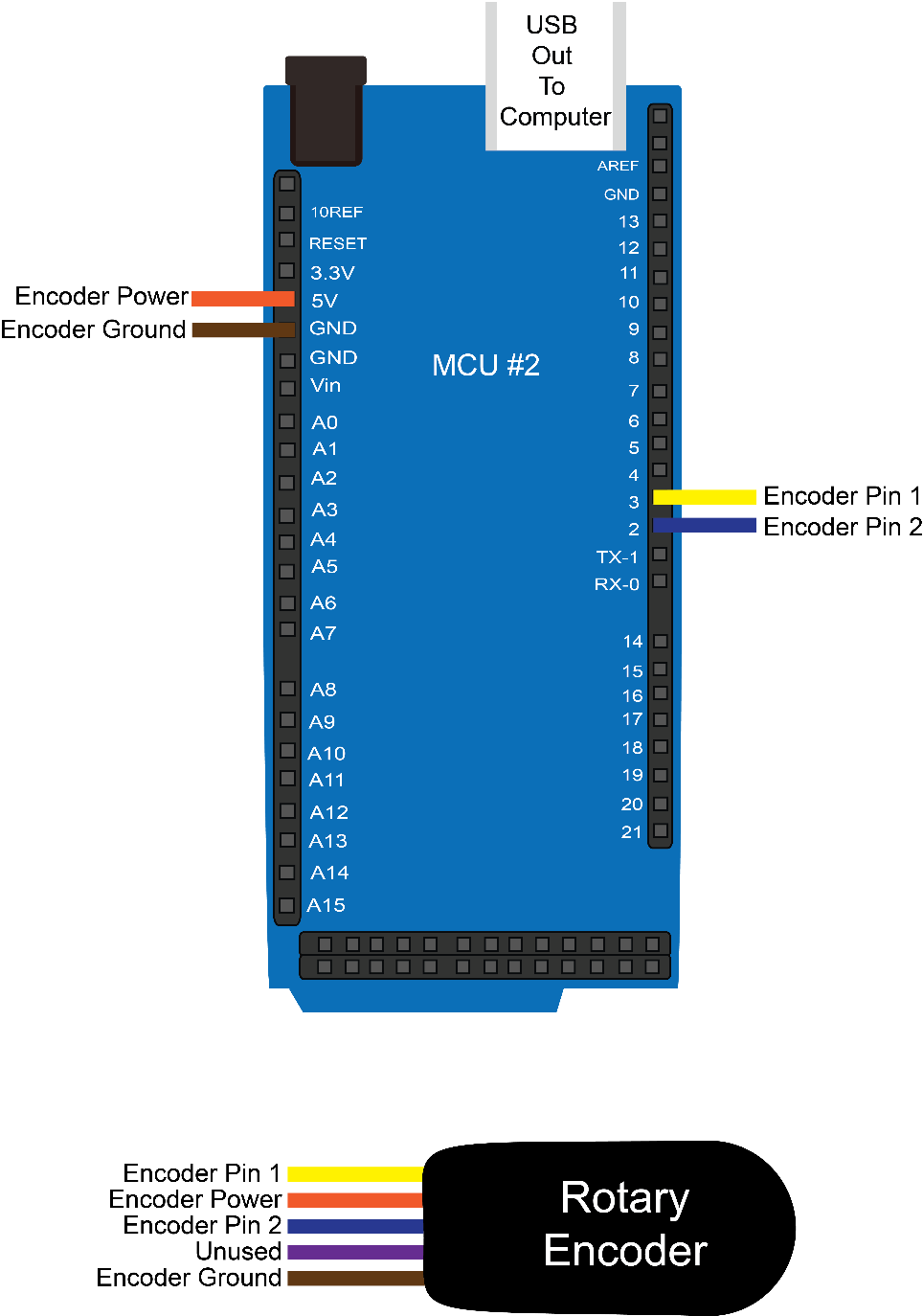


**Wiring diagram for MCU #1, capacitive touch breakouts, and relay module:**

Wires connected between MCU #2 and rotary encoder. MCU # 2 is connected to 1 component of the platform, the rotary encoder. Colors of the wires are coded to the order in which connections occur in rotary encoder, from top to bottom. Wires connected between these two components are to power the rotary encoder (orange), to ground the rotary encoder (brown), and two wires to detect the degree and direction of rotation.

Top Side 1 Side 2


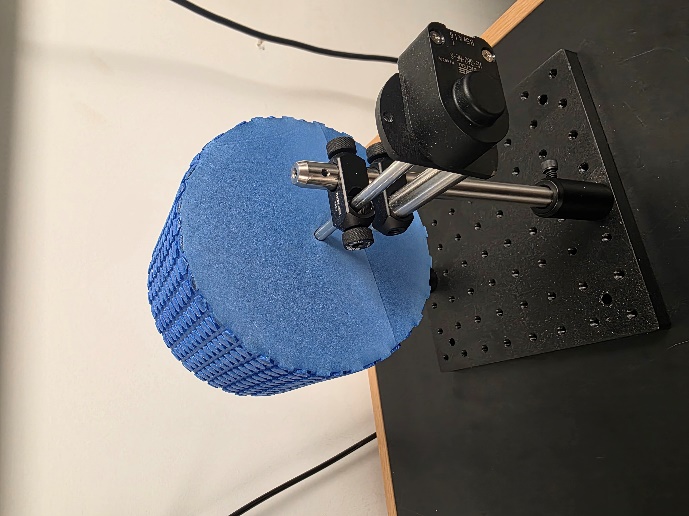

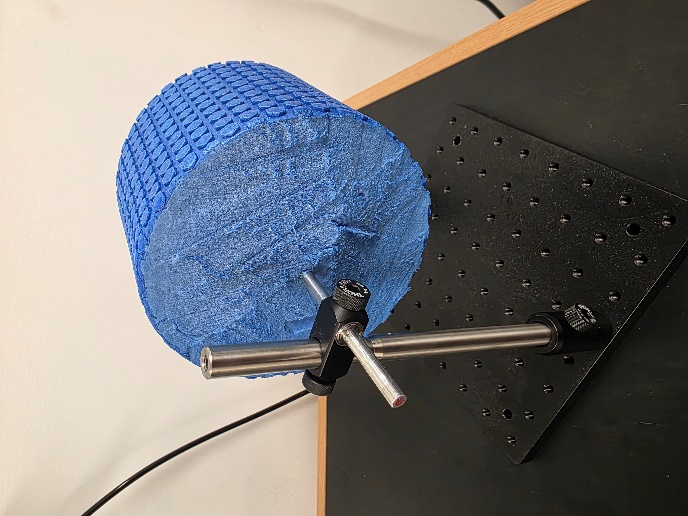


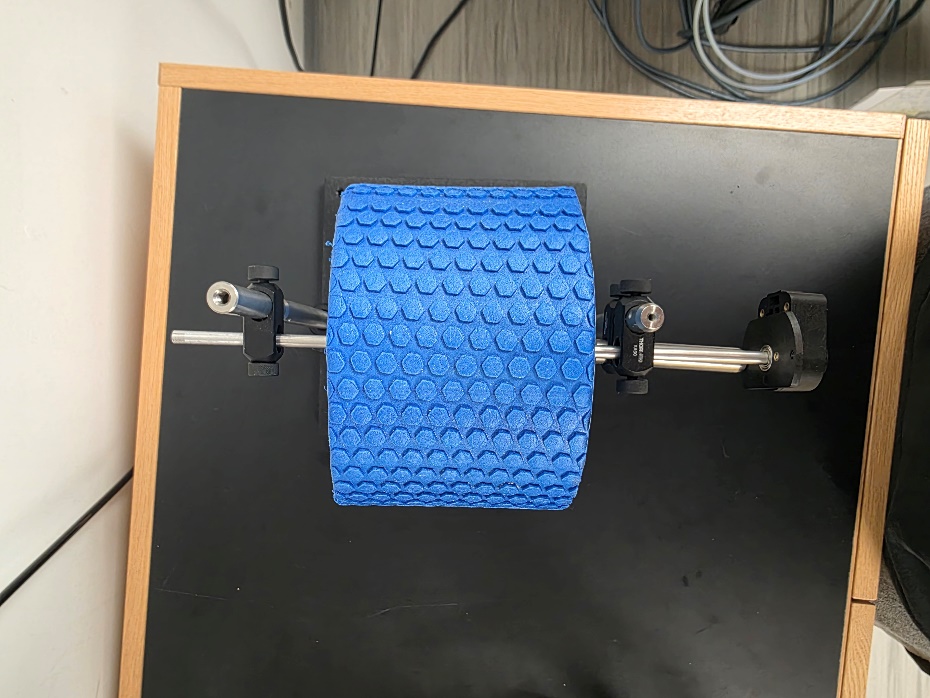


Rotary

Encoder

**Wheel assembly:** To assemble the rotary encoder into the wheel, the foam 6 in. diameter foam roller was cut into 4 in. width wheels. After wheel cutting, a hole is drilled through the center of the wheel side, perpendicular to the circular part of the wheel. The McMaster-Carr rotary shaft is then inserted into the hole of the wheel. A ball bearing is then attached to the shaft on each side of the wheel. The rotary encoder is then assembled as described in <https://www.usdigital.com/media/npwh04vh/e5-assembly-instructions.pdf>. Next, the encoder is attached to the end of the one side of the shaft, to ensure that movement on the wheel is properly recorded by the rotary encoder. The rotary encoder is connected to MCU # 2 via the cable (US Digital, part **CA-FC5-W4-NC-x**, where x is length of cable in feet)**.** Finally, the wheel is supported using the Thorlabs parts described in the parts list.

## MCU # 3 – Pupil tracking and uncoupled relay


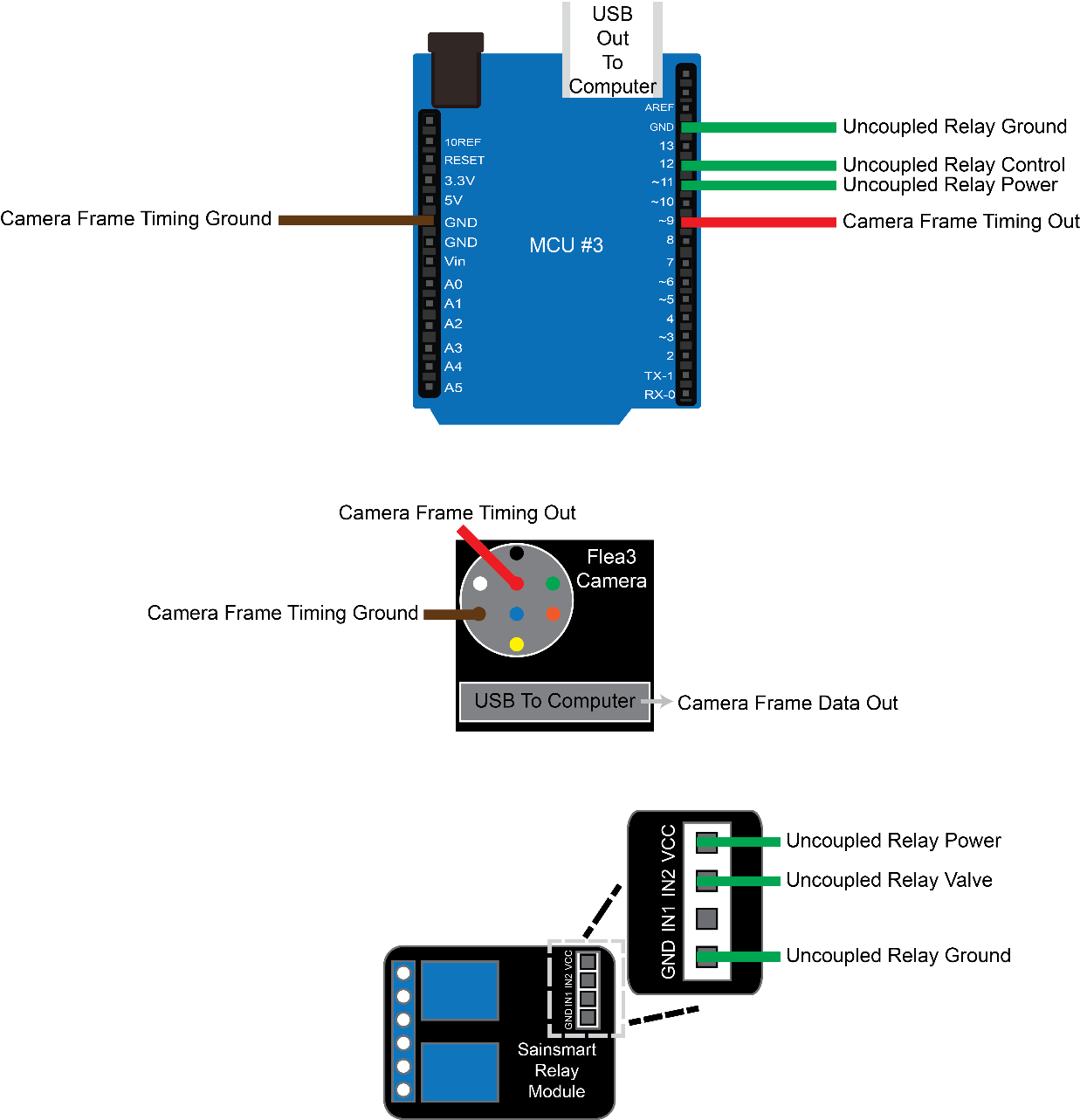


**Wiring diagram for MCU #3, camera and uncoupled relay module:**

Wires connected to MCU #3. MCU # 3 is connected to 2 components of the platform. The first component is the camera, connected via a ground wire (brown) and a TTL pulse via the frame timing wire (red). While the timing of each frame is mediated by MCU #3, the frame is directly saved to the computer via a USB connection between the camera and the computer. The second component is the uncoupled relay, connected as in MCU #1 but does not have any wires connecting the relay to a water delivery circuit.

# Freely moving luminance discrimination task:


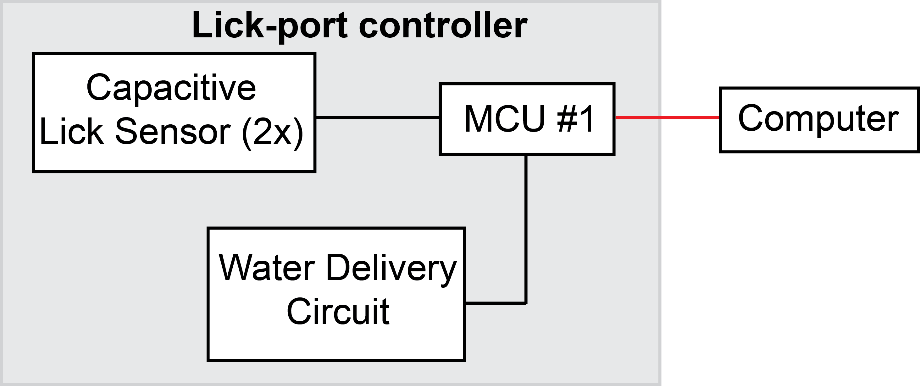


**Overview of components:**

Overview of the connections between the computer, the 1 MCU used in the freely moving luminance discrimination task and the lick-port components including the capacitive lick sensors for lick detection and the water delivery circuit for reward administration. The connection in red indicates communication between components via USB, while connections in black indication communication between components via digital I/O signals.

The MCU is connected to the computer and individual components of the platform utilized during the freely moving luminance discrimination task. Below are detailed wiring diagrams for the MCU and the components which communicate with the MCU.

**Important Note:** Wires are color coded between components and labeled such that each of the two labels for a given wire are where the given wire is supposed to be connected on each end. For example, in MCU #1, the yellow wires indicate connections between MCU #1 and the relay module, while the label “Water Valve Control Power” indicates a wire should be connected on one side to MCU #1 pin 11 and on the other side the VCC pin on the relay module.

## MCU # 1 – Lick-port controller


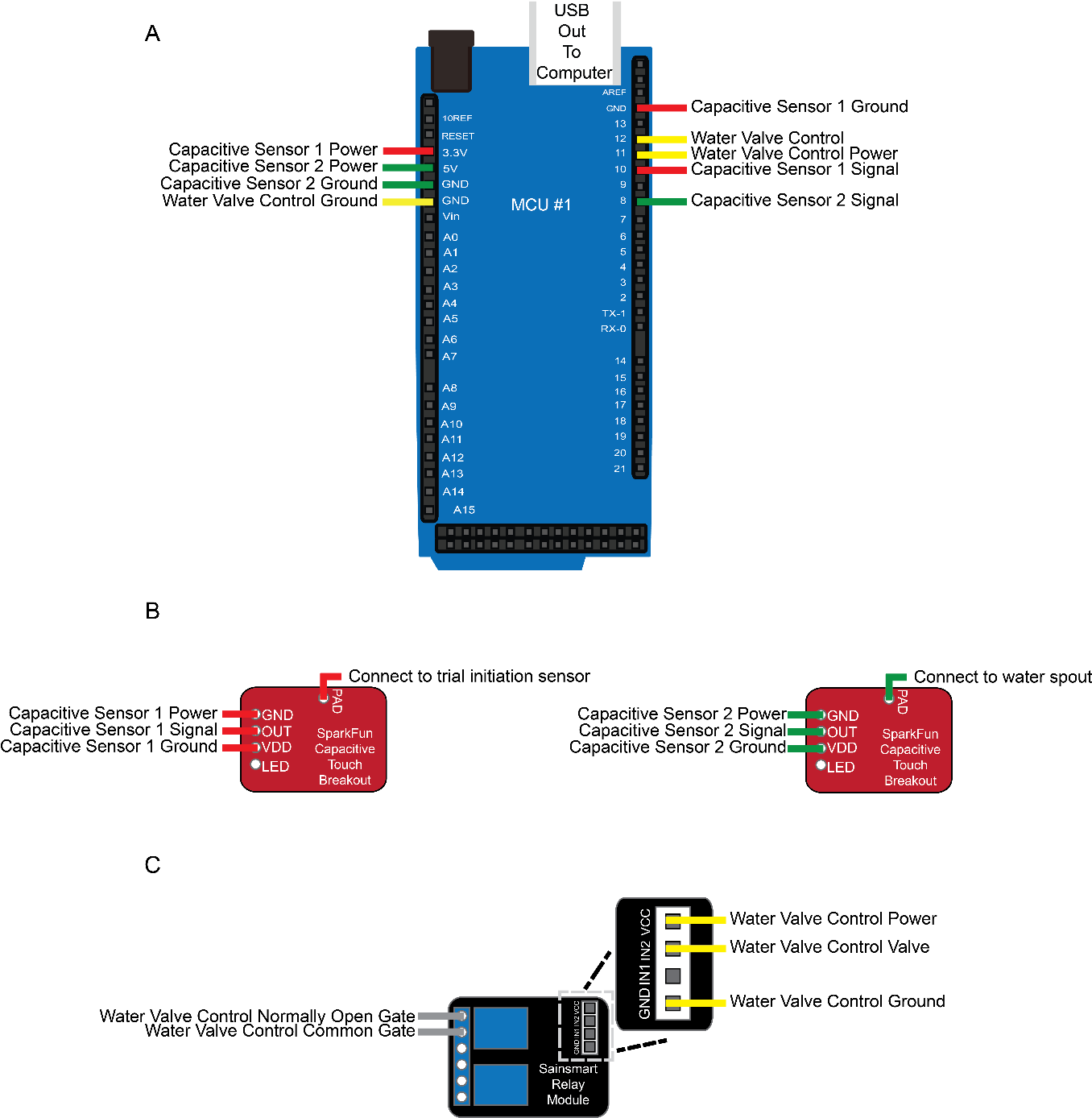


**Wiring diagram for MCU #1, capacitive touch breakouts, and relay module:**

1. Wires connected to MCU #1. MCU # 1 is connected to 3 components of the platform. Two Sparkfun capacitive touch breakouts, shown in red wire for breakout 1 and green wire for breakout 2, and the Sainsmart relay module, shown in yellow wire.
2. Wires connected to Sparkfun capacitive sensors. Each sensor receives 3 wires from MCU #1, a wire for powering the component, a wire for grounding the component, and a wire for outputting touch detections to MCU #1. In addition, each breakout has a wire connected to the PAD section, which is directly connected to the desired spout or sensor, to allow the breakout to detect touches of the spout or sensor. Capacitive sensor 1 should be connected to the trial initiation sensor and capacitive sensor 2 should be connected to the water spout.
3. Wires connected to Sainsmart relay module. Two wires, shown in gray, are connected to the relay using the upper two pins for the relay. This causes the relay to be in open state normally, preventing water flow unless when triggered. Triggering is caused by 3 wires from MCU #1 connected to the module, shown in yellow (right). From MCU #1 there is a wire to power the module, a wire to ground the module, and a wire to trigger reward delivery through the lick-port. The relay wires are connected to the water delivery circuit to cause this reward delivery, which is detailed below.


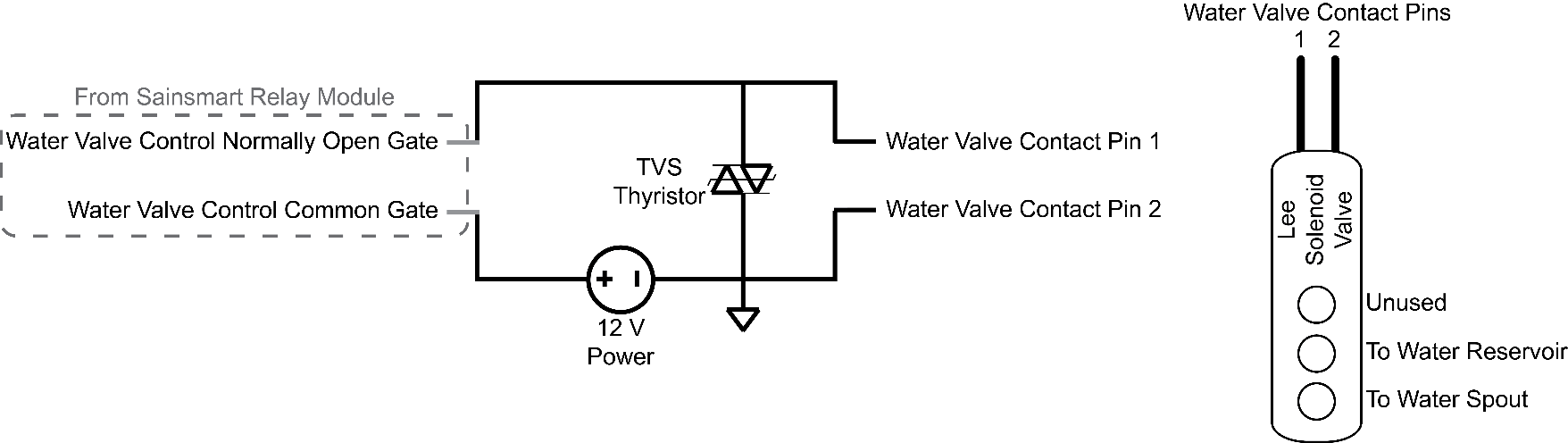


**Circuit diagram for water delivery circuit:**

A water delivery circuit is created for each lick-port (left). For the freely moving luminance discrimination task one water delivery circuit will be created. The water delivery circuit consists of 4 elements: the Sainsmart relay module, responsible for gating water delivery timing, the Lee solenoid valve, responsible for delivering water from reservoir to the metal water spout, the 12 V power supply, responsible for powering the solenoid, and the TVS thyristor, to prevent inductive kickback and protect against power surges. The Lee solenoid valve (right) is connected to the other components of the water delivery circuit via two contact pins located on the top of the valve. To facilitate water delivery, the water reservoir is connected to the center valve port, and the rubber tubing connected to the back of the lick-port is connected to the bottom valve port. This ensures that water flow is prevented, unless reward delivery is signaled.

# User Notes:

- To ensure proper communication between each MCU and the visual stimulation computer check the COM port of each MCU in device manager and modify which COM port is associated with each MCU in the MATLAB visual stimulation code.
- To ensure proper control of water delivery, ensure that the height between the bottom of the water reservoir and the end of the lick-port is 20 cm.
- If using the camera Teledyne FLIR, FL3-U3-13E4C-C which allows color, ensure the removal of the default infrared blocking filter, to allow infrared light to be recorded. Alternatively, Teledyne FLIR, FL3-U3-13E4M-C can be used, which is monochrome only and does not have this filter.

# Parts List

Behavioral Parts:

Arduino uno – Arduino, A000066

Arduino mega – Arduino, A000067

SparkFun capacitive touch breakout – SparkFun, AT42QT1011

Sainsmart relay module – Digikey; 4411-TS0010D-ND

Lee solenoid valve – Lee Company; LHDA1233115H

Water spout holder - Custom 3d printed

Hex Standoff – Digikey; 36-1921D-ND

TVS thyristor – STMicroelectronics, TPA270

12V Power Adapter – DigiKey; TRG1512-A-01E03 VI

Breadboard-friendly 2.1mm DC barrel jack – Adafruit; Product ID: 373

Metal water spout – McMaster-Carr, 6100K441

Plastic water tubing – Tygon, E-3603

Pin connectors – Mouser electronics, Mouser # 571-826629-3

Jumper wires – Mouser electronics, Mouser # 474-PRT-12796

Monitor – Dell; 30”, 2560×1600 resolution

Free behavior box – Brightroom, Bathroom Container with Lid, Qtip Holder, 6"x6"x6" Plastic

Thor labs posts – TR10, TR3, RA90, PH2

Camera parts:

Pupil camera – Teledyne FLIR, FL3-U3-13E4C-C

Camera connection cable – Flea3/Grasshopper3 8-pin GPIO Hirose Connector Cable, 1m, Edmund optics, Catalog # 88-059

Camera to Computer USB cable - Type-A to Micro-B, USB 3.1 Locking Cable, 3m, Edmund optics, Catalog # 86-770

Camera lens - Lens C125-1620-5M, Mouser electronics, Mouser # 405-2000034834

Camera IR lens – Thorlabs, part # FGL780M

Camera IR lens adapter – Thorlabs, part # SM1A25

IR light source - 850 nm light, option used for publication: <https://www.amazon.com/Univivi-Illuminator-Infrared-Security-Cameras/dp/B075F7NV56/ref=sr_1_3_sspa?crid=2OH842Y6J3LLN&keywords=univivi%2Binfrared%2Barray&qid=1742323917&sprefix=univivi%2Binfrared%2Barray%2Caps%2C78&sr=8-3-spons&sp_csd=d2lkZ2V0TmFtZT1zcF9hdGY&th=1>

Wheel parts:

Foam roller – ER-EVA-3002BD

McMaster-Carr rotary shaft – ¼” diameter, carbon steel, 12” long (part number: 1327K66)

Ball bearings – McMaster-Carr, R4-2Z (60355K43)

Rotary encoder – US Digital, part E5-1250-250-IE-S-H-R-3

Rotary encoder cable – US Digital, part CA-FC5-W4-NC-x, where x is length of cable in feet

Thor labs parts – 1x 8”-by-8” plate, 2x TR10, 1x TR3, 3x RA90, 2x PH2
