# Supplementary figures and images for: Open-Source Platform for Adjustable Training Regimes in Freely Moving and Head-Fixed Mice
Source: eNeuro. 2026 Mar 10;13(3):ENEURO.0459-25.2026. doi: 10.1523/ENEURO.0459-25.2026 (PMC13045870; doi:10.1523/ENEURO.0459-25.2026)

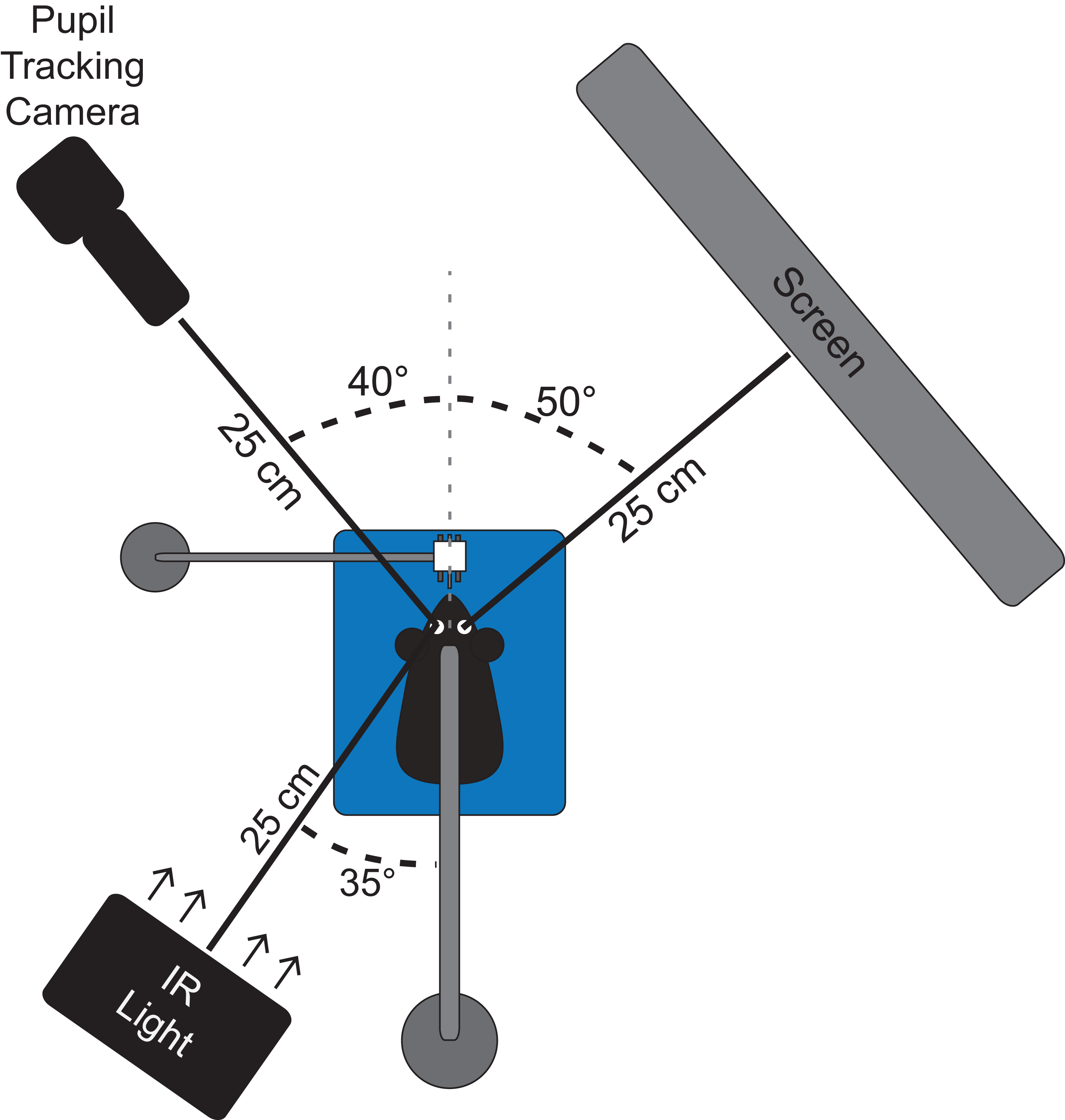

Supplement: Figure 2-1 — Illustration of the head-fixed behavioral station, top-down view. Distances (black lines of the visual stimulus screen and pupil recording camera relative to the mouse. The center of the pupil camera and screen were aligned to the height of the eyes. IR light source can be moved to best illuminate the pupil; as performed, IR light was 5 cm lower than the mouse and angled up 10° relative to horizontal to illuminate the pupil (this angle not shown). Lick-port is shown as a white square, suspended over the wheel (blue) by a rod (gray, horizontal line). Head-fixed mouse is held in position by a bar (gray, vertical line). Dashed gray line indicates the midline of the mouse. Arrows indicate direction of IR illumination. Download Figure 2-1, TIF file. [file eneuro-13-ENEURO.0459-25.2026-s002.tif]

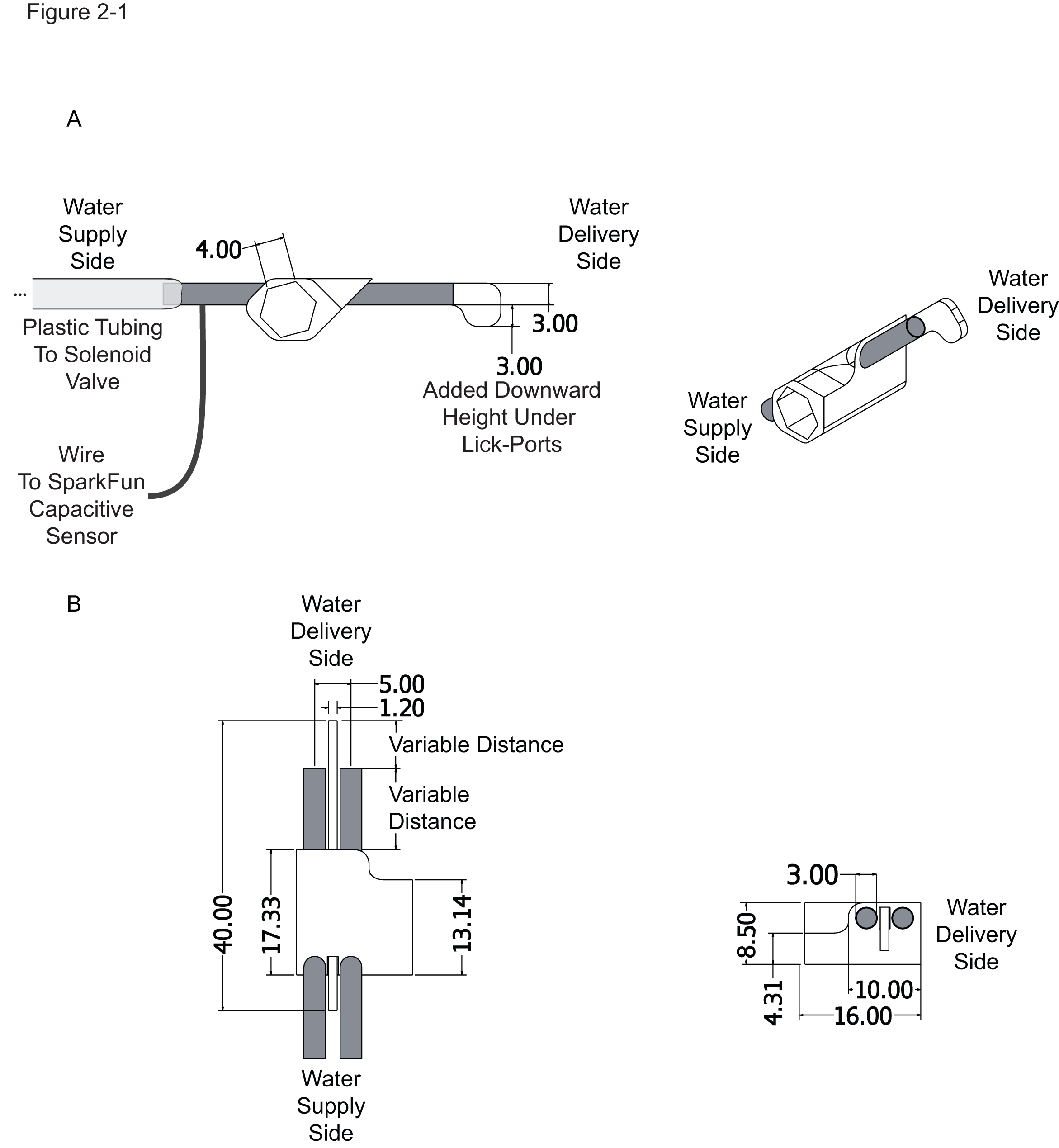

Supplement: Figure 2-2 — Dimensions of the 2 lick-port holder and divider. A) Dimensions from the side view (left) and isometric view (right) of the 2 lick-port holder and divider in relation to two water tubes (gray). The front of the divider (water delivery side) features an added downward height. This feature creates a barrier such that for each lick trajectory (which is characterized as an upward motion, starting near the lower jaw) is isolated to one side. Connection location for the plastic tubing as well as the capacitive sensor to the metal water tube shown (left). B) Dimensions from the top view (left) and front view (right) of the 2 lick-port holder and divider in relation to two water tubes (gray). The distance that the divider protrudes from the water tubes on the water delivery side can be adjusted as needed. All dimensions are reported in mm. Download Figure 2-2. Dimensions of the 2 lick-port holder and divider., TIF file. [file eneuro-13-ENEURO.0459-25.2026-s003.tif]
